# Daily Rhythmic Behaviors and Thermoregulatory Patterns Are Disrupted in Adult Female MeCP2-Deficient Mice

**DOI:** 10.1371/journal.pone.0035396

**Published:** 2012-04-16

**Authors:** Robert G. Wither, Sinisa Colic, Chiping Wu, Berj L. Bardakjian, Liang Zhang, James H. Eubanks

**Affiliations:** 1 Division of Genetics and Development, Toronto Western Research Institute, University Health Network, Toronto, Ontario, Canada; 2 Division of Fundamental Neurobiology, Toronto Western Research Institute, University Health Network, Toronto, Ontario, Canada; 3 University of Toronto Epilepsy Research Program, University of Toronto, Toronto, Ontario, Canada; 4 Department of Physiology, University of Toronto, Toronto, Ontario, Canada; 5 Department of Medicine (Neurology), University of Toronto, Toronto, Ontario, Canada; 6 Department of Surgery (Neurosurgery), University of Toronto, Toronto, Ontario, Canada; 7 Department of Electrical and Computer Engineering, University of Toronto, Toronto, Ontario, Canada; University of Insubria, Italy

## Abstract

Mutations in the X-linked gene encoding Methyl-CpG-binding protein 2 (*MECP2*) have been associated with neurodevelopmental and neuropsychiatric disorders including Rett Syndrome, X-linked mental retardation syndrome, severe neonatal encephalopathy, and Angelman syndrome. Although alterations in the performance of MeCP2-deficient mice in specific behavioral tasks have been documented, it remains unclear whether or not MeCP2 dysfunction affects patterns of periodic behavioral and electroencephalographic (EEG) activity. The aim of the current study was therefore to determine whether a deficiency in MeCP2 is sufficient to alter the normal daily rhythmic patterns of core body temperature, gross motor activity and cortical delta power. To address this, we monitored individual wild-type and MeCP2-deficient mice in their home cage environment via telemetric recording over 24 hour cycles. Our results show that the normal daily rhythmic behavioral patterning of cortical delta wave activity, core body temperature and mobility are disrupted in one-year old female MeCP2-deficient mice. Moreover, female MeCP2-deficient mice display diminished overall motor activity, lower average core body temperature, and significantly greater body temperature fluctuation than wild-type mice in their home-cage environment. Finally, we show that the epileptiform discharge activity in female MeCP2-deficient mice is more predominant during times of behavioral activity compared to inactivity. Collectively, these results indicate that MeCP2 deficiency is sufficient to disrupt the normal patterning of daily biological rhythmic activities.

## Introduction

Mutations in the X-linked gene encoding Methyl-CPG-binding protein 2 (*MECP2*) cause the neurodevelopmental disorder Rett syndrome [1], and *MECP2* mutations and duplications have been documented in several other neurodevelopmental and neuropsychiatric disorders, such as X-linked mental retardation syndrome, severe neonatal encephalopathy, Angelman's syndrome, and in some cases of idiopathic autism [2]–[4]. Further, diminished levels of MeCP2 have been noted in the autistic brain [5], and in cases of nonspecific neuropsychiatric disorder [6]. These observations highlight the essential role played by MeCP2 in establishing and maintaining neural homeostasis, and illustrate that modest alterations in its prevalence are sufficient to induce neurological impairments.

To better elucidate how MeCP2 regulates neural development and neural function, and to allow for preclinical translational studies, several mutant mouse models have been developed that either lack MeCP2 or express a clinically relevant mutant form of MeCP2 [7]–[11]. Studies in these mice have confirmed that MeCP2 deficiency alters normal brain development, synaptic communication, and neural network activities [12]–[13], and several behavioral impairments have been identified that likely stem from these neural deficiencies. To date, however, the primary behavioral parameters examined tend to rely on tests that take the subjects out of their cages, and transiently expose them to a new environment for assay. While these tasks have high value for assessing specific behavioral endpoints, the daily cyclic behavioral performance of the mutant mice in their home environment is not typically assessed, and electrographic brain wave activity patterns that are known to correlate with specific behaviors in wild-type subjects remain largely uninvestigated in MeCP2-deficient mice. Given the apparent link between impaired MeCP2 function and altered behavioral state rhythmicity in Rett syndrome patients [14] and in *Mecp2^308/y^* mice [15], we sought to determine whether impaired MeCP2 function would be sufficient to alter the daily EEG behavior, thermoregulatory, and/or periodic ambulatory cycles of mice. Here, we provide the first report of how these daily cyclic activity patterns are affected by a heterozygous deficiency of *Mecp2* in female mice.

## Materials and Methods

### Ethics Statement

All animal experimentation was conducted in accordance with the guidelines of the Canadian Council of Animal Care, and thoroughly reviewed and approved before implementation by the Toronto General and Western animal care committee (Protocol 1321.7). All surgery was performed under general anesthesia, and every effort was made to minimize suffering.

### Animal Subjects

Two strains of MeCP2-deficient mice (*Mecp2^tm1.1Bird^* [7] and *Mecp2^tm2Bird^* [16], obtained from Jackson Laboratories, Bar Harbor, ME) were used in this study. The *Mecp2^tm1.1Bird^* (n = 7), *Mecp2^tm2Bird^* (n = 4), and wild-type mice were all female, aged between 300 and 400 days, and maintained on a pure C57Bl/6 background. Although different in molecular design, the *Mecp2* gene is disrupted in each of these lines, and each displays common phenotypic progression [16]. Genotyping was done via polymerase chain reaction (PCR) as described previously [16]–[17]. All animals were housed in a vivarium that was maintained at 22–23°C with a standard 12-hour light on/off cycle commencing at 6:00. For this study, Zeitgeber time of 0 refers to the 6:00 lights-on daily time.

### Implantation Surgery

Experimental mice were implanted with a mouse-specific wireless telemetry probe (TA11ETA-F10; Data Sciences International (DSI), St. Paul, MN) for recording of body temperature, general activity and EEG. The surgical implantation procedure was as described previously [18] with minor modifications. Briefly, mice were anesthetized with 2% isoflurane and the wireless transmitter placed into their peritoneal cavity. Silicone elastomer insulated sensing and reference wires connecting the transmitter were orientated rostrally toward the head via a subcutaneous route. The sensing wire was soldered to an intracranial EEG polyimide-insulated stainless steel electrode with an outside diameter of 125 µm, and placed in the parietal cortex region (bregma −0.6 mm, lateral 1.5 mm, and depth 1.5 mm) with the reference wire placed at bregma −5 mm, lateral 1 mm, and depth 1.5 mm. The implantation surgery caused no apparent abnormalities in the mice, and average body weights of both *Mecp2^−/+^* and wild-type mice returned to pre-operative values within 2 weeks post-surgery (32.3 g versus 32.9 g and 26.8 g versus 27.0 g for *Mecp2^−/+^* (n = 11) and wild-type (n = 8) respectively).

### Electrophysiology Data Collection

Body temperature, activity, and EEG waveforms were collected from the implanted mice for continuous 24-hour periods. Waveform data was transmitted from the TA11ETA-F10 telemetry probes to a wireless receiver (RPC-1, DSI), which passes the data through a data exchange matrix serving as a multiplexer (DSI), and was analyzed using DataQuest A.R.T. (DSI). Body temperature was acquired using the TA11ETA-F10's thermosensor from the peritoneal cavity. Gross locomotive activity was determined by assessing the standard deviation of the wireless signal strength of the transmitter in relationship to two receiving antennae arranged perpendicularly in the RPC-1 wireless receiver. This method and arrangement has been used previously to track and measure locomotive activity in mice [19]–[20]. The accuracy of the system to detect ambulatory movement was further validated by visually comparing the activity output of the system with movement revealed by synchronized video recordings. Analysis of random 10 minute segments from these video data revealed that the collection program detected all of the ambulatory movements in the mice and conversely that >95% of the activity identified by the program was accompanied by visible gross movement by the mouse (n = 5 mice, Video S1). Both temperature and motor activity data were transmitted at a rate of 50 Hz, using a sampling frequency (analog to digital) of 250 Hz. The EEG waveform was transmitted at 200 Hz and sampled at 1 kHz.

### Characterization of cortical epileptiform discharge events

24 hour EEG traces were visually inspected to confirm and quantify the presence of discharge activity as described previously [20]–[21]. In brief, a discharge event was defined as having amplitudes of at least 1.5-fold background, durations of at least 0.4 seconds, and a frequency of between 6 and 10 Hz. Two genotype-blinded investigators independently assessed EEG activity, and the individual counts averaged. The overall concordance between these individuals was 86.4%, and these differences were averaged for final analysis of discharge incidence rate and the times of discharge occurrence over the 24-hour cycle. Having confirmed the presence of discharge activity using established manual criteria [20]–[21], we then developed an automated method to characterize the duration and frequency components of the discharges. For this, a 6–10 Hz FIR band pass filter was applied to specifically isolate the frequency band associated with the discharges. The envelope of the filtered signal was produced by convolution of the square of the filtered data with a Gaussian kernel of 200-point aperture [22]. This envelope peaks whenever strong 6–10 Hz activity is present. As normal cortical EEG signals rarely display high-amplitude rhythmic spiking within this frequency, the envelop peak reflects discharge events (Figure S1). To determine discharge durations, the left and right inflection points of detected events were used to find the start and end points respectively. The inflection points were computed by convolving the envelope with the derivative of the Gaussian kernel as above. The DataQuest A.R.T. program (DSI) was used to generate total spectral plots over the 24-hour period for individual mice. Time-frequency analysis was conducted using the continuous wavelet transform (CWT) found in the Matlab digital signal processing toolbox. The basis function used in the CWT analysis was the Morlet mother wavelet [23]–[24], which is commonly used in EEG analyses [23]. To minimize the issue of scaling, the analysis was divided into low frequencies (0.5–30 Hz) and high frequencies (30–80 Hz), with 0.5 Hz step size.

### Recognition of periodic variations in EEG, gross motor activity, and core body temperature

EEG signals within the delta band (0.5–4 Hz) [25] were extracted by applying a series of steps. First, the data were pre-processed by removing segments indicative of movement artifacts (characterized by voltages higher than 0.5 V), and the 0.25 second time period preceding and succeeding these events. Then, a FIR band pass filter with an order of 1000 was applied to isolate specifically the delta band. Delta power was obtained by squaring the delta band signal, and then averaging these values over 30 second intervals so the resulting value aligns with the movement activity and temperature signals (which were also recorded at 30 second sampling periods) by the data acquisition system. The Pearson's product-moment correlations between delta power, motor activity, and core body temperature were conducted using smoothed versions of these raw 30 second interval data. The smoothing function employed was the 50-point Fast Fourier Transformation (FFT) in OriginPro 6.1 (OriginLab Corporation, Northampton, MA). To then discern the daily patterning of these three signals, each was normalized to have 0 mean and variance of 1, and a Gaussian-based kernel with aperture 50 was applied on all three signals generating an envelope of the signals. A threshold of 0 was then applied to discretize the signals into two different states (Figure S2). The delta power parameter was discretized into delta and non-delta states with a ‘complete delta cycle’ being defined as a state of delta followed by state of non-delta, where each individual state has a duration of at least 15 minutes. Similarly, ‘mobility cycles’ and ‘body temperature cycles’ were defined as the combination of a consecutive active and an inactive state, or consecutive high body temperature and a low body temperature states, respectively.

### Statistical analysis

Student's t-tests were used for direct comparisons between two groups. For comparisons between multiple groups, one-way ANOVA with Bonferroni post hoc correction for multiple comparisons was utilized. F-tests were used to compare the equality of two variances between groups. For comparing the correlative strength between two groups, Pearson's product moment correlation coefficient, a determiner of linear dependency between two variables, was employed. Significance was set at p<0.05. Mean and standard error of the mean are presented throughout the text and figures.

## Results

### The general properties of cortical EEG activity are preserved in *Mecp2^−/+^* mice

**Figure 1:**
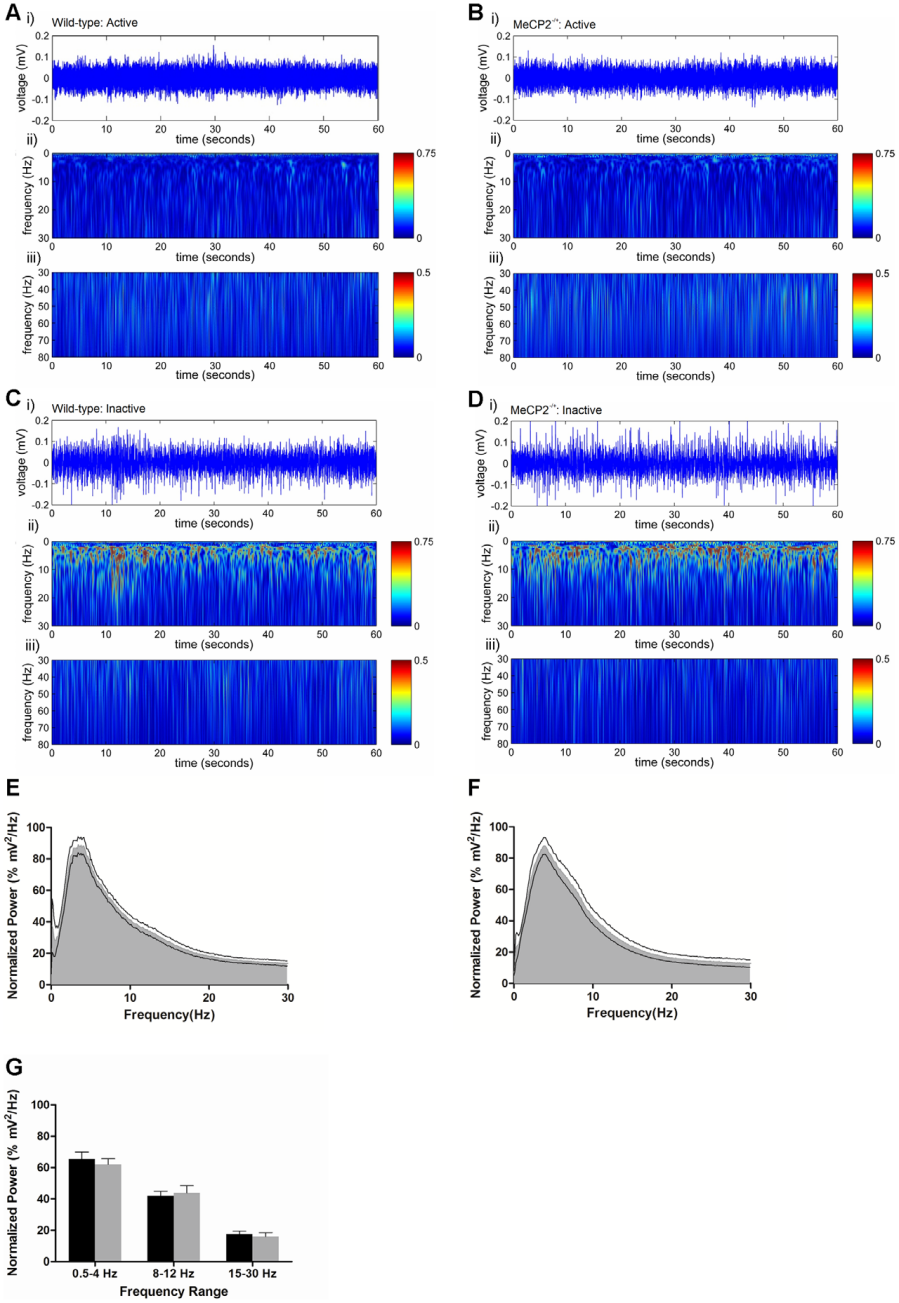
The general EEG waveform properties of the *Mecp2^−/+^* mouse cortex are similar to wild-type. Panels A–D: Representative examples of a 1 minute segment of raw EEG activity (i) taken from wild-type (A and C) and *Mecp2^−/+^* mice (B and D) during mobility (A and B) and during inactivity (C and D). Shown below each raw EEG trace is the corresponding wavelet transformation showing the spectrum of frequency power for the 0.5–30 Hz range (ii) and for the 30–80 Hz range (iii). Panels E and F: Average normalized time-frequency power spectrum plots of wild-type (E) and *Mecp2^−/+^* (F) mice. Panel G: Average power of the delta band (0.5–4 Hz), the alpha band (8–12 Hz) and beta band (15–30 Hz) in wild-type and *Mecp2^−/+^* mice normalized to peak power for each respective mouse. Histograms are plotted as mean ± SEM. Asterisks denote statistical significance *p*<0.05, student's unpaired t-test for n = 11 *Mecp2^−/+^* mice and n = 8 wild-type mice.

Consistent with our previous observations [21], the general neocortical EEG signals in adult *Mecp2^−/+^* mice did not display overt differences from adult female wild-type mice. Waveforms with elevated amplitude and slow frequency (0.5–4 Hz, delta band) were evident during immobile and sleep-like behavior, while lower amplitude higher frequency activity was seen during periods of movement or exploration. CWT time-frequency analyses [23] of these cortical EEG activities (excluding periods of EEG discharge activity, see below) revealed no qualitative differences in the frequency powers of wild-type and *Mecp2^−/+^* mice within the 0.5–80 Hz spectrum during either the active or inactive states of behavior (Figure 1A–D). Additionally, time-frequency analysis of *Mecp2^−/+^* and wild-type 24-hour EEG waveforms using a Fast Fourier Transformation revealed the overall power spectrum distributions between the two groups was preserved (Figure 1E, F), and analysis of specific waveforms, e.g., the delta band (0.5–4 Hz), alpha band (8–12 Hz) and beta band (15–30 Hz), across the full 24-hour day also revealed no significant differences in overall power between the groups (Figure 1G).

### *Mecp2^−/+^* mice display alterations in their daily pattern of cortical delta wave activity

**Figure 2:**
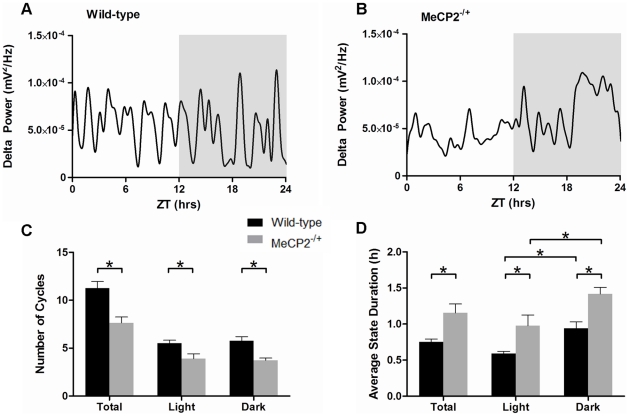
*Mecp2^−/+^* mice display altered daily delta cycle periodicity and duration. Panels A and B: Representative traces of delta power patterning over 24 hours in a wild-type (A) and a *Mecp2^−/+^* (B) mouse. The dark phase (ZT 12–24) is shaded in these plots. Panels C and D: Histograms showing the average number of delta cycles (C), and the average duration of time spent in a non-delta state per delta cycle (D). For panels C and D, the total 24-hour data set is shown with the data for either the light (ZT 0–12) or dark (ZT 12–24) phases specifically. Shown are the mean ± SEM for n = 11 *Mecp2^−/+^* and n = 8 wild-type mice. Asterisks denote statistical significance at *p*<0.05, one-way ANOVA with Bonferroni *post hoc* correction for multiple comparisons.

The presence of delta slow wave cortical EEG waveforms is often used as an indicator of sleep in wild-type rodents [26]. Analysis of smoothed cortical delta power (as derived from the EEG waveforms, Figure S3A–D) in wild-type mice revealed clearly defined patterns of rhythmicity over the 24-hour period (Figure 2A). In contrast, the daily patterns of delta power in *Mecp2^−/+^* mice were more erratic in periodicity and duration (Figure 2B). Comparison of wild-type and *Mecp2^−/+^* mice revealed a significant decrease in the average number of delta cycles over a 24-hour period (12 hours light, 12 hours dark, Figure 2C). *Mecp2^−/+^* mice displayed an average of 7.6±0.6 delta cycles compared to 11.3±0.7 in controls (*p* = 0.001). This overall decease was equivalently diminished in both phases of the 24-hour day (*p*<0.01, for each respectively, Figure 2C). Further, the average duration of the non-delta state of each cycle period was significantly longer in *Mecp2^−/+^* mice than wild-type mice (1.5±0.3 hours versus 0.75±0.1 hours, *p*<0.05, Figure 2D). This increase in non-delta state duration was present during both the light and dark phases of the 24-hour day, and consistent with the preferential nocturnal behavior of mice, both *Mecp2^−/+^* and wild-type mice displayed greater non-delta time durations in the dark phase of the 24-hour day.

### *Mecp2^−/+^* mice display alterations in daily cyclic mobility patterns

**Figure 3:**
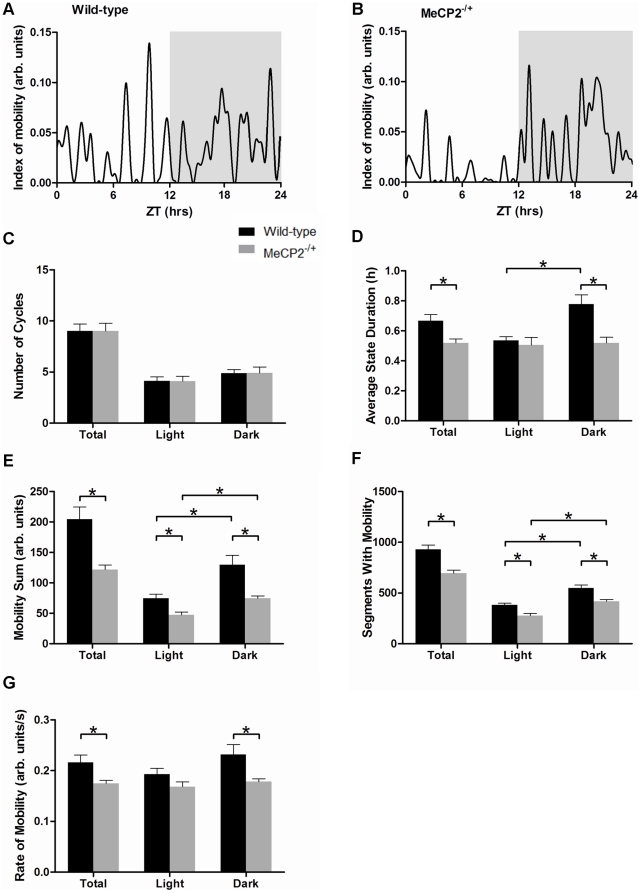
*Mecp2^−/+^* mice display abnormal cycles of daily activity and mobility deficits in their home cage environment. Panels A and B: Representative traces of activity patterning over 24 hours in a wild-type (A) and a *Mecp2^−/+^* (B) mouse. The dark phase is shaded. Panels C and D: Histograms showing the average number of mobility cycles (C), and the average duration of time spent in an active state per mobility cycle (D). For panels C and D, the total 24-hour data set is shown with the results specific for either the light or dark phases. Panels E–G: Histograms showing the home-cage activity parameters of *Mecp2^−/+^* and wild-type mice over the full 24 hours, and for the light and dark phases of the day specifically. Panel E shows the total amount of mobility of mice over 24 hours. Panel F shows the number of 30-second segments throughout the day in which *Mecp2^−/+^* and wild-type mice displayed mobility. Panel G shows the average rate of movement (magnitude of movement per second) performed by *Mecp2^−/+^* and wild-type mice. Shown are the mean ± SEM for n = 11 *Mecp2^−/+^* and n = 8 wild-type mice. Asterisks denote statistical significance at *p*<0.05, one-way ANOVA with Bonferroni *post hoc* correction for multiple comparisons.

In contrast to the alterations in delta power periodic patterning, examination of smoothed mobility patterns (Figure S3E–H) failed to reveal differences in cycle number between wild-type (Figure 3A) and *Mecp2^−/+^* mice (Figure 3B) over the 24-hour day. *Mecp2^−/+^* mice displayed an average of 9.0±0.8 total mobility cycles over the 24-hour day, while wild-type mice displayed an average of 9.0±0.7 cycles (Figure 3C). However, although the number of mobility cycles was preserved, the distribution of time in the active phase versus the inactive phase of these cycles differed between *Mecp2^−/+^* and wild-type mice. Specifically, the average duration of the active state of a cycle was significantly decreased in *Mecp2^−/+^* mice over the 24-hour day (0.52±0.03 hours versus 0.66±0.04 hours, *p*<0.05, Figure 3D), with the difference being predominant in the dark phase of the 24-hour cycle. Wild-type mice showed longer active state durations in the dark relative to the light phases (0.78±0.06 hours versus 0.54±0.02 hours in dark and light respectively, *p*<0.005), while *Mecp2^−/+^* mice exhibited similar active state durations in both light and dark phases (0.52±0.04 hours versus 0.50±0.05 hours in dark and light respectively, Figure 3D).

### *Mecp2^−/+^* mice possess altered home cage mobility profiles

The reduced active state duration in *Mecp2^−/+^* mice suggests that they spend more time in the awake-immobile state than the awake-active state relative to wild-type mice. Analysis of the raw movement profiles revealed a significant reduction in the total amount of mobility (as deduced by changes in strength of the telemetry signal at the receiver) between *Mecp2^−/+^* mice and wild-type mice (121±7 versus 204±20 mobility counts, respectively *p*<0.005). Further, the overall time spent by *Mecp2^−/+^* mice moving in their home cages over a 24-hour period was significantly reduced compared to age-matched wild-type mice (694±31 versus 931±43 segments containing mobility, *p*<0.001, Figure 3E, F). In addition to total mobility differences, the average rate of movement by the *Mecp2^−/+^* mice was also diminished relative to wild-type (0.17±0.01 versus 0.22±0.01 mobility counts/sec, *p*<0.05), and this effect was the most pronounced in the dark phase of the day (Figure 3G).

### The inverse correlation of delta power and behavioral activity is disrupted in *Mecp2^−/+^* mice

**Figure 4:**
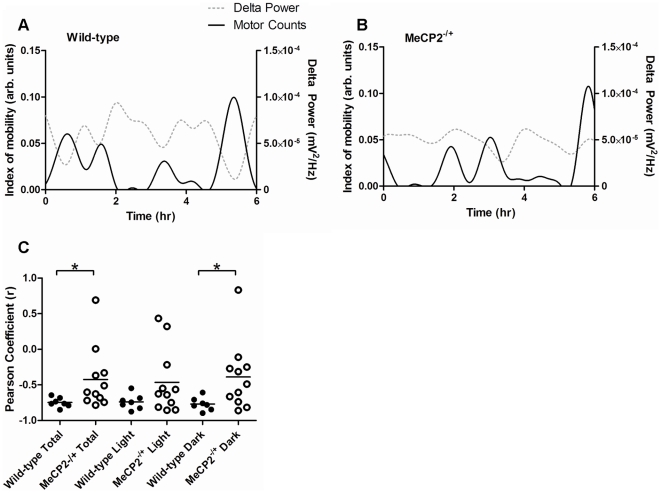
The normal correlation between cortical delta power and mobility is altered in *Mecp2^−/+^* mice. Panels A and B: Representative traces of activity and delta power parameters over a 6 hour period in the light phase of the day for a wild-type (A) and a *Mecp2^−/+^* (B) mouse. For each, the solid black line denotes mobility while the grey dotted line denotes delta power. Panel C: Scatter plots showing the Pearson's product-moment correlation coefficients for delta power compared to activity in *Mecp2^−/+^* and wild-type mice. Each point represents the daily correlative strength for a single subject. The bar on the scatter plot indicates the mean for each set. Asterisks denote statistical significance (p<0.05) between the indicated groups (student's unpaired t-test). n = 11 *Mecp2^−/+^* and n = 7 wild-type mice, respectively.

In wild-type mice, there was a strong inverse correlation between mobility and cortical delta power (Figure 4A). This was not the case in *Mecp2^−/+^* mice (Figure 4B). The strength of the Pearson's product-moment correlation coefficient (a measure of linear dependency) revealed a strong inverse correlation (average r = −0.75) between delta power and movement in wild-type mice, consistent with delta power serving as a good predictor for sleep/immobility in wild-type mice [26]. However, the Pearson's correlation coefficient for delta power and movement in *Mecp2^−/+^* mice was significantly weaker (average r = −0.42), indicating that delta power is not a good predictor for immobile or sleep states in *Mecp2^−/+^* mice (Figure 4C). In fact, as shown in Figure 4B, instances of high delta power concomitant with mobility were frequently observed in *Mecp2^−/+^* mice.

### *Mecp2^−/+^* mice display impaired body temperature patterning and regulation

**Figure 5:**
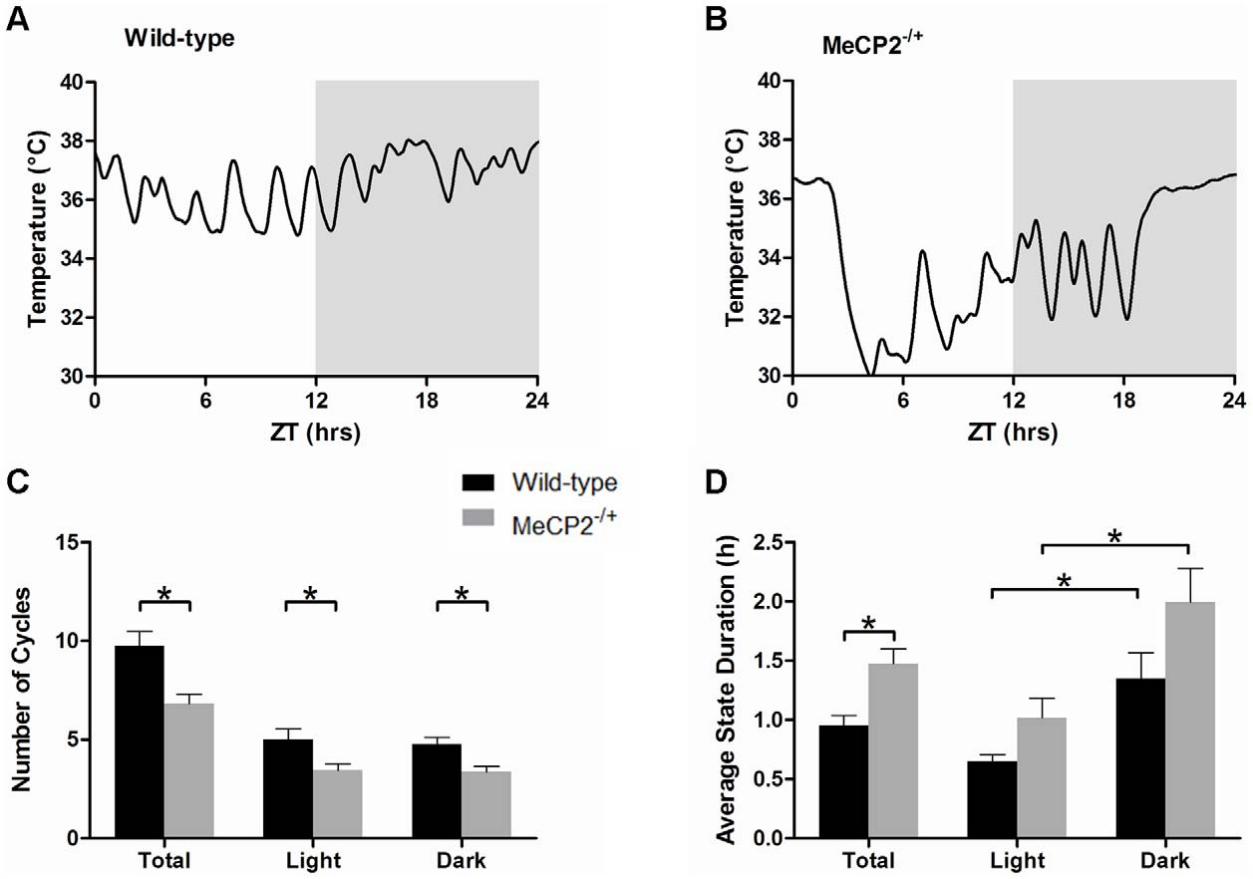
The normal pattern of body temperature cycling is altered in *Mecp2^−/+^* mice. Panels A and B: Representative traces of daily body temperature over 24 hours in a wild-type (A) and a *Mecp2^−/+^* (B) mouse. The dark phase of the day is shaded on each panel. Panels C and D: Histograms showing the average number of body temperature cycles (C), and the average duration of time spent in the top half of the full temperature range for each mouse across the day (D). For panels C and D, the total 24-hour data set is shown along with the results specific for either the light or dark phases. Shown are the mean ± SEM for n = 11 *Mecp2^−/+^* and n = 8 wild-type mice. Asterisks denote statistical significance at *p*<0.05, one-way ANOVA with Bonferroni *post hoc* correction for multiple comparisons.

**Figure 6:**
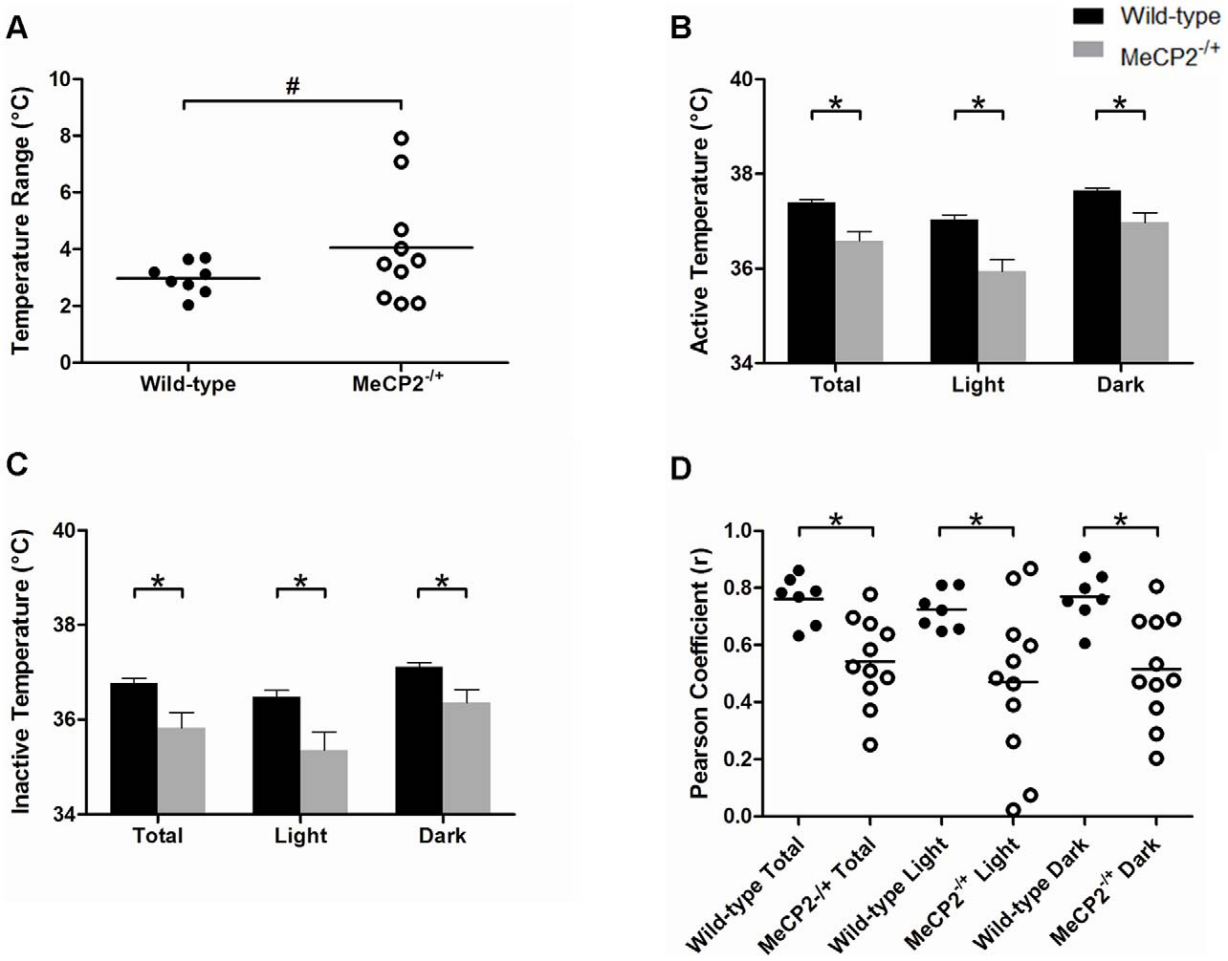
Core body temperature regulation is altered in *Mecp2^−/+^* mice. Panel A: Scatter plot showing the range of core body temperature in *Mecp2^−/+^* and wild-type mice. Each point represents the absolute range (min to max during the 24-hour day) of core body temperature for an individual mouse. On Panel A, # denotes p<0.05 as determined using an F-test for the equality of two variances. Panel B and C: Histograms showing the mean ± SEM of the active body temperature of *Mecp2^−/+^* and wild-type mice (B), and their average inactive body temperature (C) throughout the day, or specifically during the light or dark phases. Panel D: Scatter plot showing the Pearson's product-moment correlation coefficients for mobility and temperature in *Mecp2^−/+^* and wild-type mice. Each point represents the daily correlative strength between mobility and temperature for a single subject. The bar on the scatter plot indicates the mean for each set. Asterisks denote statistical significance (p<0.05) between the indicated groups (student's unpaired t-test). n = 11 *Mecp2^−/+^* mice and n = 7 wild-type mice.

The patterns of cyclic body temperature fluctuations (derived from smoothed raw data, Figure S3I–L) also revealed differences between wild-type (Figure 5A) and *Mecp2^−/+^* mice (Figure 5B). *Mecp2^−/+^* mice displayed fewer temperature cycles per day than wild-type mice (6.8±0.5 versus 9.8±0.7, Figure 5C), and an increase in the average duration of time spent in the high phase of their temperature cycle relative to wild-type mice (1.47±0.13 versus 0.95±0.09 hours, respectively, Figure 5D). In addition to having impaired periodic rhythmic patterns, the average daily minimal temperature, and the average daily maximal temperature of *Mecp2^−/+^* mice were each significantly lower than wild-type (33.9±0.7°C versus 35.6±0.2°C respectively, for minimum, *p*<0.05; and 37.9±0.2°C versus 38.5±0.1°C respectively, for maximum, *p*<0.05). Consistently, the core body temperature range of *Mecp2^−/+^* mice had higher variance than wild-type mice over the 24-hour day (4.06°C^2^ range versus 0.31°C^2^ range, respectively, *p*<0.005, Figure 6A). Moreover, during periods of mobility and inactivity specifically, the temperature of *Mecp2^−/+^* mice was significantly lower than that of wild-type mice (36.6±0.2°C versus 37.4±0.1°C for mobile states and 35.8±0.3°C versus 36.8±0.1°C for inactive states, *p*<0.005 and p<0.05, Figure 6B, C), and the correlation coefficient between movement and body temperature in the *Mecp2^−/+^* mice was substantially weaker than that of the wild-type mice (0.76 versus 0.54, respectively, *p* = 0.001, Figure 6D). Collectively, these results indicate *Mecp2^−/+^* mice display an overall reduction in core body temperature throughout the day, and that their homeostatic regulation of body temperature is impaired.

### *Mecp2^−/+^* mice display spontaneous cortical epileptiform discharge activity

**Figure 7:**
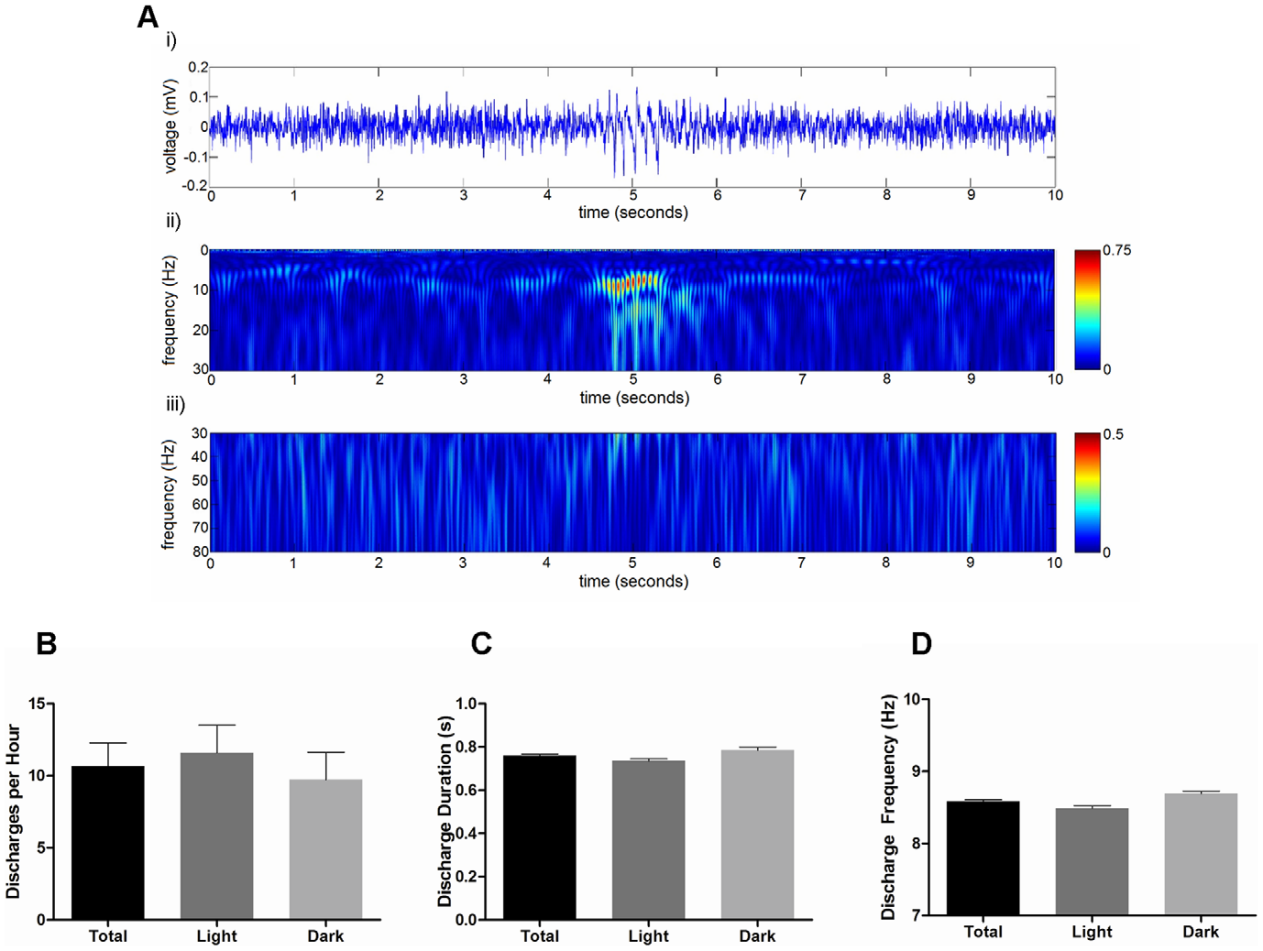
Properties of epileptiform discharges in *Mecp2^−/+^* mice. Panel A: Representative example of a 10 second segment of raw EEG activity (i) illustrating a typical discharge event in a *Mecp2^−/+^* mouse, and the corresponding wavelet transformation showing the spectrum of frequency power for the 0.5–30 Hz range (ii) and for the 30–80 Hz range (iii). Panels B–D: Histograms showing the mean ± SEM of the discharge rate per hour (B), the average discharge duration (C), and the average frequency component of all the discharges (D) in *Mecp2^−/+^* mice. Presented on each histogram is the total over the 24-hour period, and the data stratified for specifically light and dark phases. No statistically significant differences in discharge activity, duration, or frequency were seen between the light and day phases (student's paired t-test, n = 8 *Mecp2^−/+^* mice).

Raw EEG waveform data was examined from *Mecp2^−/+^* mice to determine the prevalence and distribution of epileptiform discharges throughout the 24-hour period. For these assessments, a discharge event was defined as a high amplitude rhythmic waveform lasting at least 0.4 seconds with a frequency between 6 and 10 Hz (Figure 7A). No discharge activity was detected in any of the wild-type mice examined (n = 8). Cortical EEG discharges were observed in 8 of 11 *Mecp2^−/+^* mice. In these mutants, the average number of cortical epileptiform discharges per hour over a 24-hour period was 10.7±1.6 (Figure 7B). The average duration of the discharge events was 0.76±0.01 seconds, and the average frequency of the discharges was 8.6±0.02 Hz (Figure 7C, D). While spontaneous convulsions were not observed in any of the *Mecp2^−/+^* mice, cortical discharge activity was associated with behavioral freezing, which often lasted longer than the duration of the discharge (data not shown). Analysis of discharge activity during the light and dark phases of the day failed to reveal any significant differences: the incidence rate of the discharges, their average duration, and their average frequency did not significantly differ during the light or dark phases.

### Cortical epileptiform discharge activity predominates during the active state of a mobility cycle

**Figure 8:**
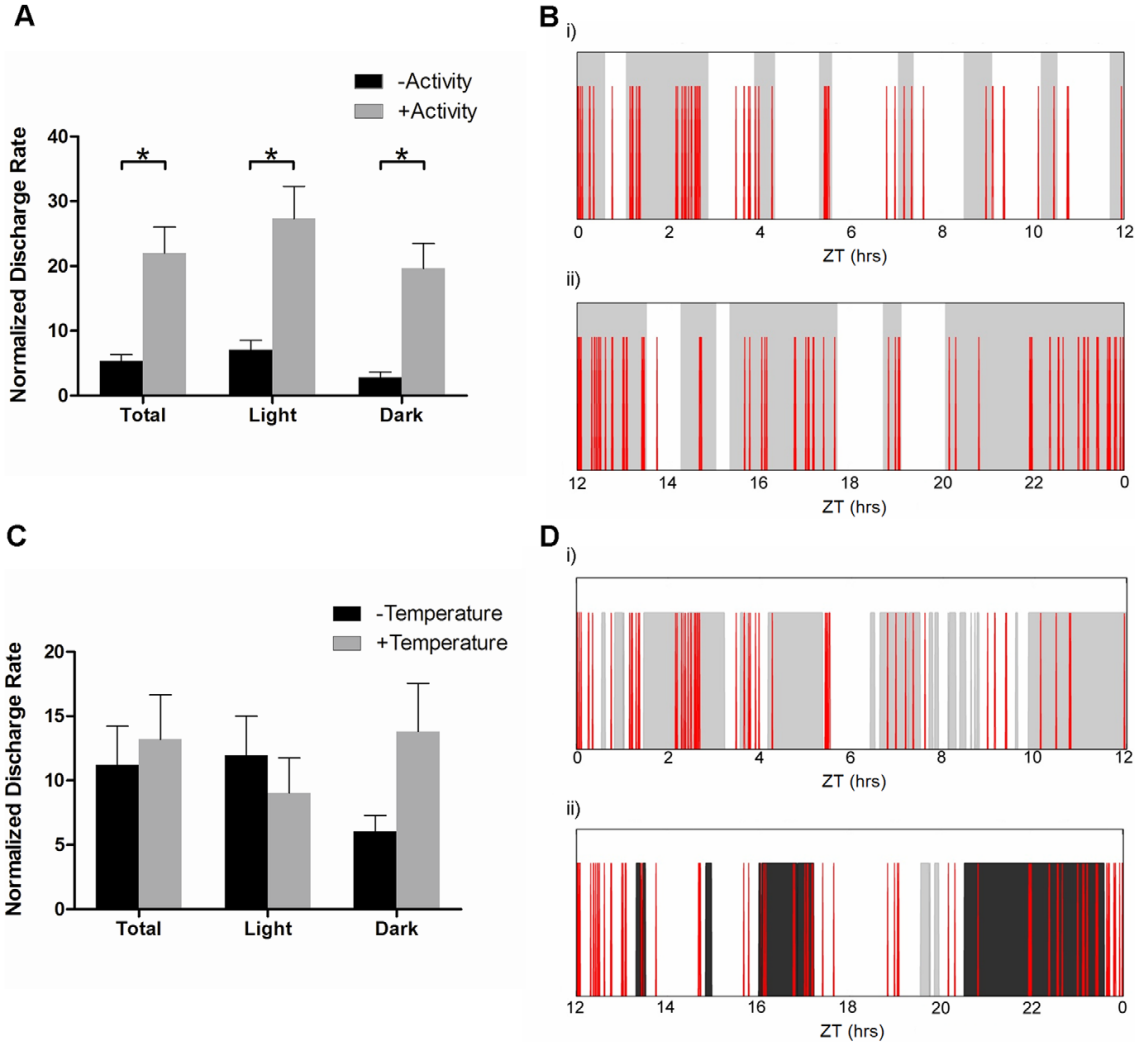
Epileptiform discharge activity in *Mecp2^−/+^* mice differs between behavioral states. Panels A and B: Incidence rate of cortical discharge activity during either the mobile or inactive behavioral states. The histogram (A) shows the mean ± SEM of the discharge rate per hour, normalized to the time spent in each behavioral state as above. Panel B shows a representative plot of epileptiform discharge distribution over the light (i) and dark (ii) phases of a 24-hour day. Red spikes represent individual discharge events and the shaded regions denote times in which mobility was present. Panels C and D: Incidence rate of cortical discharge activity when core body temperature for the *Mecp2^−/+^* mice was within the top 25% (high) or the lowest 25% (low) of the mean value for their 24-hour cycle. The histogram (C) shows the mean ± SEM of the discharge rate per hour, normalized for time spent in each temperature category as above. Panel D shows a representative plot in the same similar format as Panels B, except dark shading reflects times when temperature was in the upper 25% and light shading reflects time when temperature was in the lower 25% of the daily range. Times spend in the intermediate temperature range show no shading. Asterisks denote statistical significance (*p*<0.05) as determined using a Student's paired *t*-test, for n = 8 *Mecp2^−/+^* mice.

To assess whether cortical discharge activity occurred randomly throughout the day, or was preferentially seen during certain behavioral states, we compared discharge activity across active and inactive states, and in periods of high and low core body temperature. These assessments revealed that significantly higher discharge activity was found in *Mecp2^−/+^* mice during the active phase of their behavioral mobility cycle during the entire day (22.0±4.0 versus 5.4±1.0 discharges, *p*<0.005, Figure 8A, B), and during the light and dark phases of the day, specifically. In contrast, no significant association between core body temperature and discharge activity was seen. For this, we compared discharge activity in mice during times when their core body temperature was within the top or bottom 25% range of the full 24-hour day. No significant differences in discharge rate were observed between these periods of high and low core body temperature either during the entire day (13.2±3.5 versus 11.2±3.0 discharges per hour, respectively *p* = 0.63, Figure 8C, D), or during the light or dark phases specifically.

## Discussion

In this study, we examined the daily periodic cortical EEG waveform activity, body temperature, and movement activity parameters of *Mecp2^−/+^* mice in their home-cage setting. Five principal observations emerge from our work. First, the normal daily pattern of cyclic EEG delta wave activity is altered in *Mecp2^−/+^* mice. These mutants display a decreased number of daily delta cycles, and spend longer periods than normal in a low delta power state. Second, *Mecp2^−/+^* mice display significantly less movement in their home-cage environment, particularly during the nocturnal phase, and display significantly more time in an awake-but-inactive state. Third, the daily minimum, maximum, and overall average temperature of *Mecp2^−/+^* mice is lower than that of wild-type mice. Fourth, *Mecp2^−/+^* mice display spontaneous cortical epileptiform discharges, and this discharge activity is most pronounced when the mouse is in an active behavioral state. Fifth, the daily rhythmic and correlative patterns of delta power, movement activity, and body temperature are significantly altered in *Mecp2^−/+^* mice. Collectively, these investigations identify novel behavioral deficits associated with MeCP2 deficiency, and provide a new investigative procedure that can be employed for translational studies.

Although there is clear evidence for disrupted sleep-wake cycles in Rett syndrome patients [27]–[28], there have been few assessments of whether normal biological patterning is altered in MeCP2-deficient mice. Our data show that *Mecp2^−/+^* mice display significantly disrupted daily behavioral patterns compared to age and gender-matched wild-type mice. Specifically, *Mecp2^−/+^* mice display reduced numbers of normal cortical delta activity and body temperature cycles over a 24-hour period. High delta power has been used as an index for determining sleep and awake times in wild-type animals [26] [29]. Consistent with this, we found a strong correlation between periods of high delta power and periods of low activity in wild-type mice. Intriguingly, though, this correlation was not observed in *Mecp2^−/+^* mice, where high delta power was often observed during periods of high activity. This suggests that the normal homeostatic balance of neural circuits is disrupted in the *Mecp2^−/+^* brain. However, we cannot exclude the possibility that a movement artifact caused by the slow ambulatory patterns of MeCP2^−/+^ mice may have contributed to this signal. Irrespective of origin, the clear difference in delta power and activity correlational strength between wild-type and MeCP2^−/+^ mice illustrates a phenotypic difference that arises from the MeCP2 deficiency.

The observation of disrupted daily rhythmic patterning in *Mecp2^−/+^* mice is consistent with the recent studies that found *Mecp2* mRNA to be a direct target of the microRNA miR-132 [30]–[31]. miR-132 expression is robustly induced within neurons of the suprachiasmatic nucleus (SCN) by light stimulation [32], and miR-132 negatively regulates MeCP2 protein levels in these neurons. This regulation of MeCP2 expression is one component of the system regulating the expression of Period genes and thus contributes to clock entrainment [30]. Given the strong evidence that disruption of clock gene regulation in SCN is sufficient to alter cortical delta periodicity and power [33], the altered delta patterns we observe in *Mecp2^−/+^* mice are in line with MeCP2 playing a significant role in circadian regulation, as suggested by Alverez-Savaadra et al. [30]. Based on these results and our findings, it would be of interest to further explore whether alterations of cortical delta activity patterns occur in Rett syndrome patients and/or in patients with other MeCP2-related neural disorders.

In addition to disrupted rhythmic behavioral patterning, *Mecp2^−/+^* mice displayed diminished overall movement in their home-cage setting. Consistent with previous results from *Mecp2^308/y^* male mice [17] [34], the activity of female *Mecp2^−/+^* mice was reduced similarly during the light and dark phases of the diurnal cycle. Analysis of the home-cage body temperature also revealed alterations in daily temperature cycling patterns in *Mecp2^−/+^* mice. *Mecp2^−/+^* mice showed significant decreases in both their peak minimum and maximum body temperature over the day, and collectively showed an overall decrease in their average body temperature – both throughout the day, and also during periods of activity and inactivity specifically. These observations confirm and extend from those of a recent report in which the basal body temperature of male *MeCP2^−/y^* mice was found to be reduced compared to wild-type mice [35]. In addition to showing a decrease in average daily temperatures, though, our results also show that the range of normal body temperature fluctuation over the day is significantly greater in *Mecp2^−/+^* mice, and that *Mecp2^−/+^* mice have a poorer ability than wild-type mice to regulate body temperature. Collectively, these results are consistent with impaired autonomic nervous system function, which is a cardinal phenotype of clinical Rett syndrome.

In agreement with our previous acute study [21], we observed the presence of abnormal epileptiform-like discharges in the somatosensory cortex of *Mecp2^−/+^* mice. Our examination of the distribution of discharges throughout the 24-hour diurnal cycle revealed no differences in discharge incidence between the light and dark phases of the day. However, the incidence rate for discharge activity did correlate with times when the mutant mice were in specific behavioral states. Significantly more discharge activity was observed in the mutants during times of activity and/or movement compared to times of immobility. Perhaps surprisingly, however, no differences in discharge rate were seen in the mice when their body temperature was in the upper or lower 25% of their daily range. This result was somewhat unexpected, as lower temperature tends to slow metabolic processes, and has been linked to an attenuation of seizure rates [36]. The most likely explanation for this is that although lower, the decreased core body temperature is not sufficient to have a major effect on neural activity, and thus the hyper-excitability of the MeCP2-deficient circuits is not diminished.

In summary, in this study we conducted the first concurrent examination of 24-hour cortical EEG waveforms, movement activity, and body temperature profiles in *Mecp2^−/+^* mice in their home-cage environment. Our results indicate that in addition to attenuating home-cage movement activity, MeCP2 deficiency is sufficient to alter the normal daily cyclic patterns of cortical delta wave activity and body temperature. Further, we characterize the average incidence, frequency, and duration of epileptiform discharges in *Mecp2^−/+^* mice over a 24-hour period, and show that there is a relationship between their behavioral state and the prevalence of cortical discharge activity.

## Supporting Information

Video S1 **Synchronized video recording and activity output of a MeCP2^−/+^ mouse.** This video shows a 1 minute segment of a MeCP2^−/+^ mouse in its home cage environment. Shown below the video is the activity output plot generated by the DSI analysis program that is synchronized to the video recording. Note the concordance of the ambulation activity of the mouse with the peaks depicted in the plot.
(MOV)

Figure S1 **Automated detection of epileptiform discharges.** Panel A: Raw 10-second EEG waveform segment collected from a representative MeCP2^−/+^ mouse displaying 2 epileptiform discharges as determined and confirmed by visual inspection (Red lines represent the start and end of the respective discharge event). Panel B: Resulting envelope of the EEG waveform in Panel A after band pass filtering the signal through a 6–10 Hz FIR filter and then convoluting the square of this filtered data with a Gaussian kernel of 200 point aperture (Red lines represent the start and end of the respective discharge events, the green line represents the envelope of the black 6–10 Hz FIR band pass filtered signal). Panel C: Resulting derivative of the convolved envelope signal presented in Panel B used to determine the start and end of the discharge event. The red lines denote the left and right inflection points used to determine the start and end of the discharges, respectively.
(DOC)

Figure S2 **Recognition of periodic variations in EEG, gross motor activity, and core body temperature.** Panels A and B: Representative traces of cortical delta power patterning over the light (i) and dark (ii) phases of a 24 hour day in a wild-type (A) and a MeCP2^−/+^ (B) mouse. Shaded regions denote areas classified as high delta states and non-shaded regions denote areas classified as low (non) delta states. Panels C and D: Representative traces of mobility patterning over a 24 hour day in a wild-type (C) and a MeCP2^−/+^ (D) mouse. Shaded regions denote areas classified as mobile behavioral states whereas non-shaded regions denote areas classified as inactive behavioral states. Panels E and F: Representative traces of core body temperature patterning over a 24 hour day in a wild-type (E) and a MeCP2^−/+^ (F) mouse. Shaded regions denote areas where body temperature was above the daily mean value, whereas non-shaded regions denote areas where body temperature was below the mean.
(DOC)

Figure S3 **Illustration of smoothed data generated from raw delta power, mobility, and body temperature traces.** Panels A–D: Representative traces of raw cortical delta power (grey line) and the resulting smoothed data (black line) as generated using the 50-point Fast Fourier Transformation (FFT) smoothing function in OriginPro 6.1 (OriginLab Corporation, Northampton, MA) for a wild-type (A and B) and a MeCP2^−/+^ (C and D) mouse during the day (A and C) and night (B and D) phases of the 24-hour day. Panels E–H: Representative traces of raw mobility (grey line) and the resulting smoothed data (black line) as generated above for a wild-type (E and F) and a MeCP2^−/+^ (G and H) mouse during the day (E and G) and night (F and H) phases of a 24-hour day. Panels I–L: Representative traces of raw core body temperature (grey line) and the resulting smoothed data (black line) as generated above for a wild-type (I and J) and a MeCP2^−/+^ (K and L) mouse during the day (I and K) and night (J and L) phases of a 24-hour day.
(DOC)
