# Supplementary figures and images for: Daily Rhythmic Behaviors and Thermoregulatory Patterns Are Disrupted in Adult Female MeCP2-Deficient Mice
Source: PLoS One. 2012 Apr 16;7(4):e35396. doi: 10.1371/journal.pone.0035396 (PMC3327685; doi:10.1371/journal.pone.0035396)

**Figure S1**


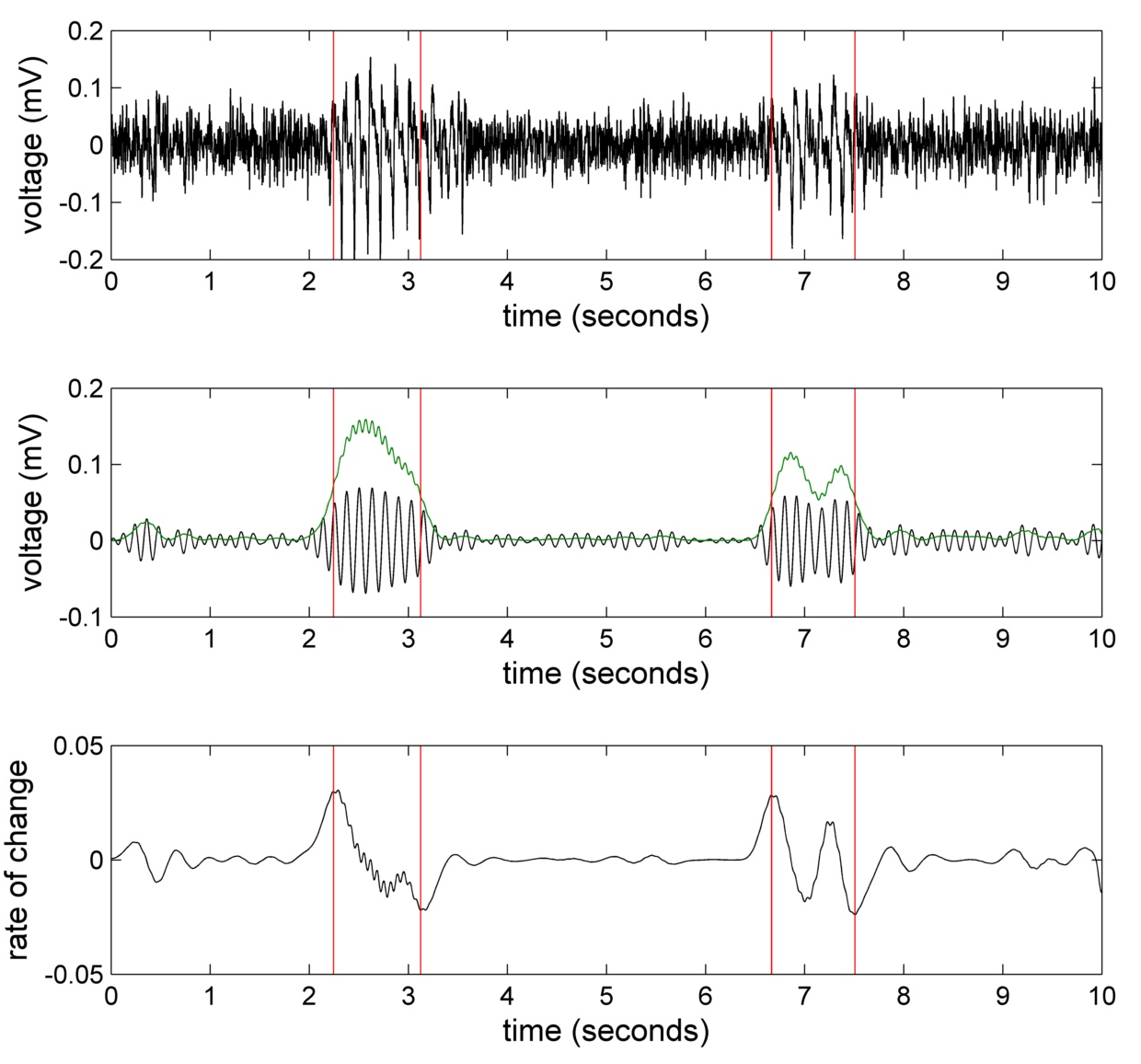
**A**

**B**

**C**

Supplement: Figure S1 — Automated detection of epileptiform discharges. Panel A: Raw 10-second EEG waveform segment collected from a representative MeCP2−/+ mouse displaying 2 epileptiform discharges as determined and confirmed by visual inspection (Red lines represent the start and end of the respective discharge event). Panel B: Resulting envelope of the EEG waveform in Panel A after band pass filtering the signal through a 6–10 Hz FIR filter and then convoluting the square of this filtered data with a Gaussian kernel of 200 point aperture (Red lines represent the start and end of the respective discharge events, the green line represents the envelope of the black 6–10 Hz FIR band pass filtered signal). Panel C: Resulting derivative of the convolved envelope signal presented in Panel B used to determine the start and end of the discharge event. The red lines denote the left and right inflection points used to determine the start and end of the discharges, respectively. (DOC) [file pone.0035396.s002.doc]

**Figure S2**


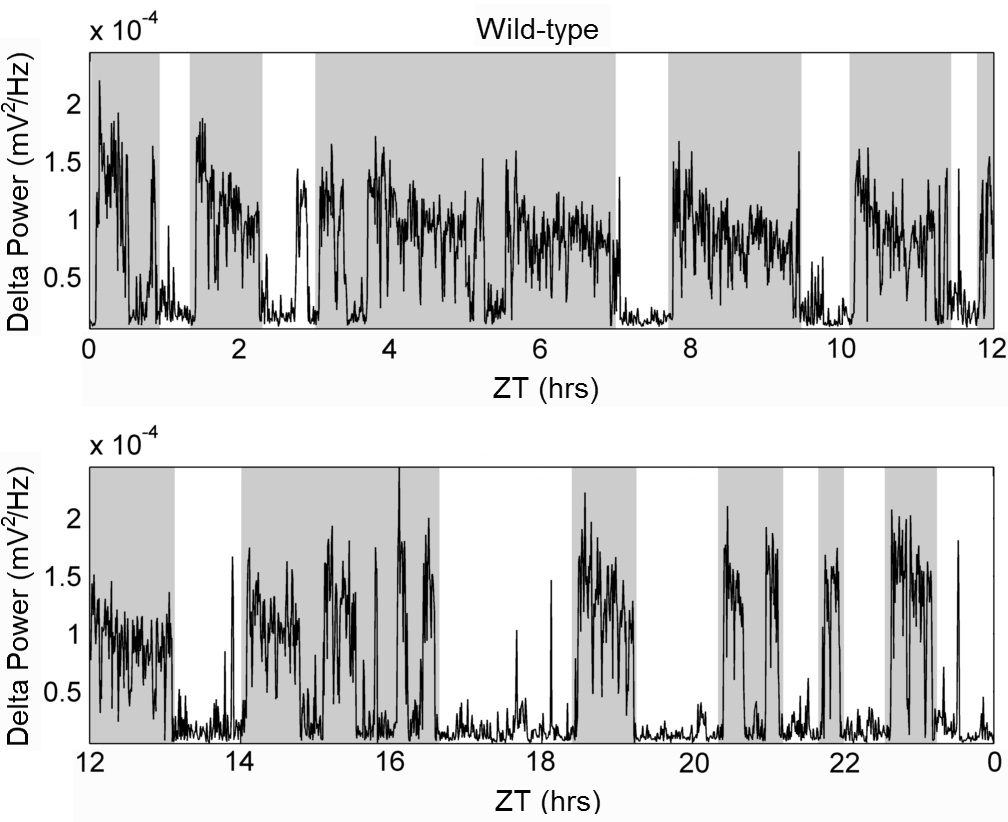
**A i)**

**ii)**


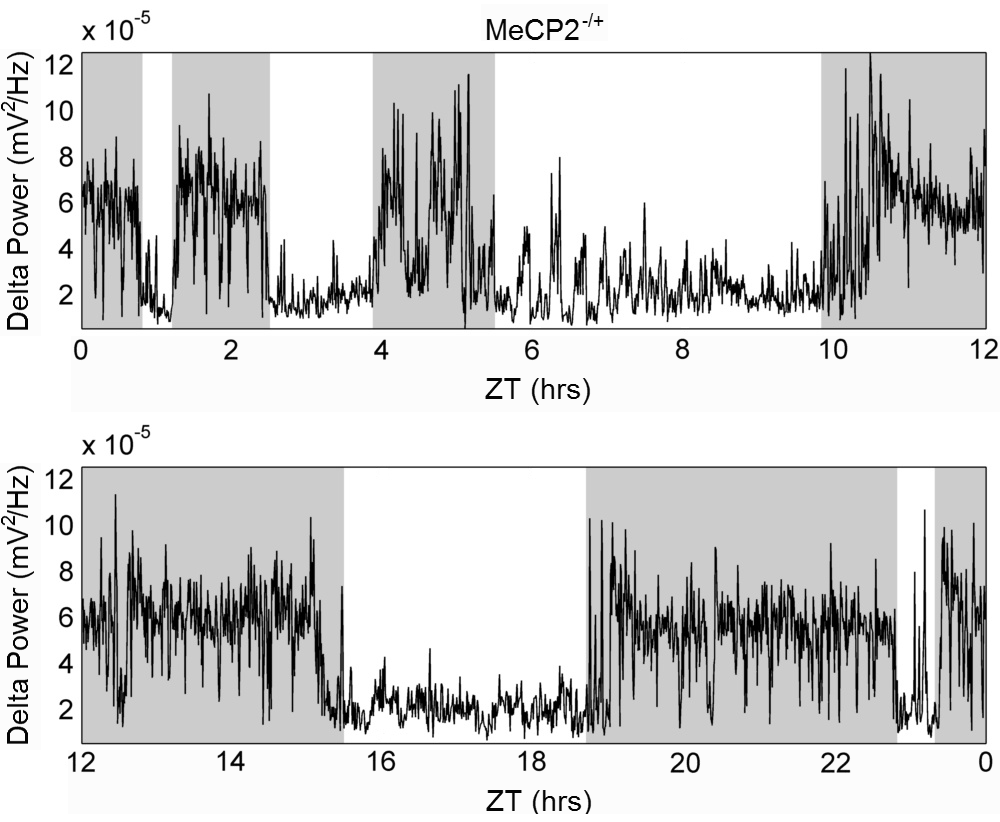
**B i)**

**ii)**

**Figure S2 Cont’d**

**C i)**


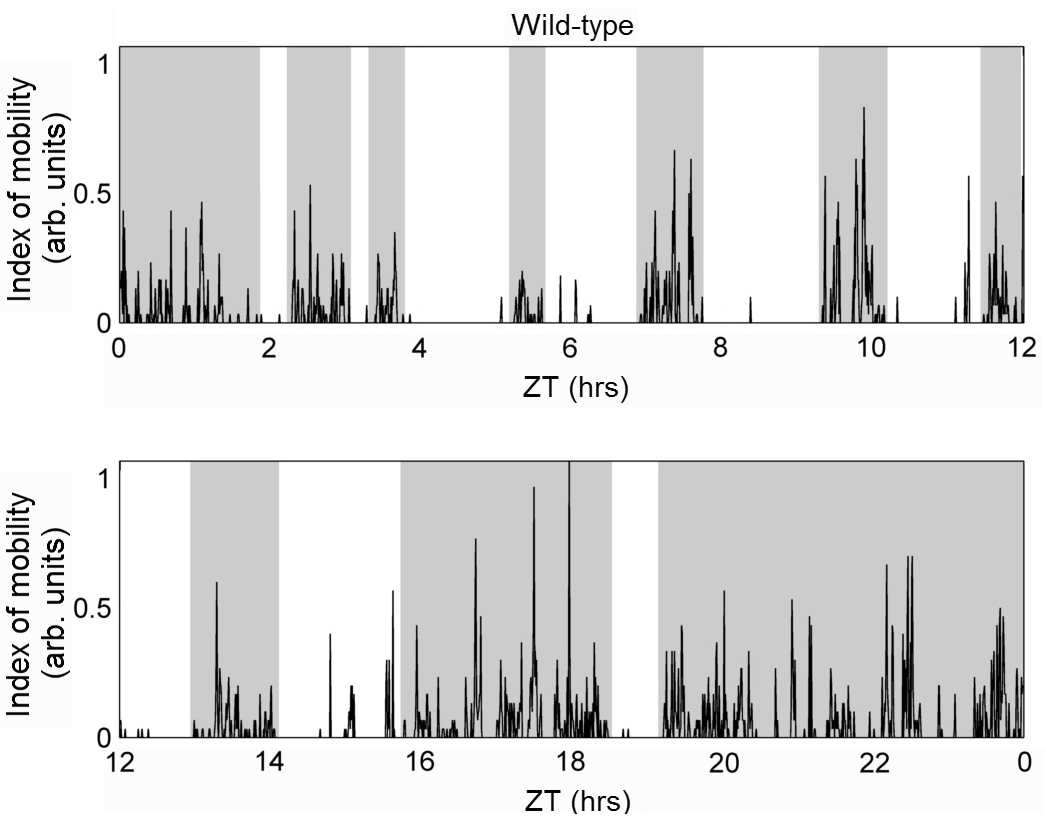


**ii)**


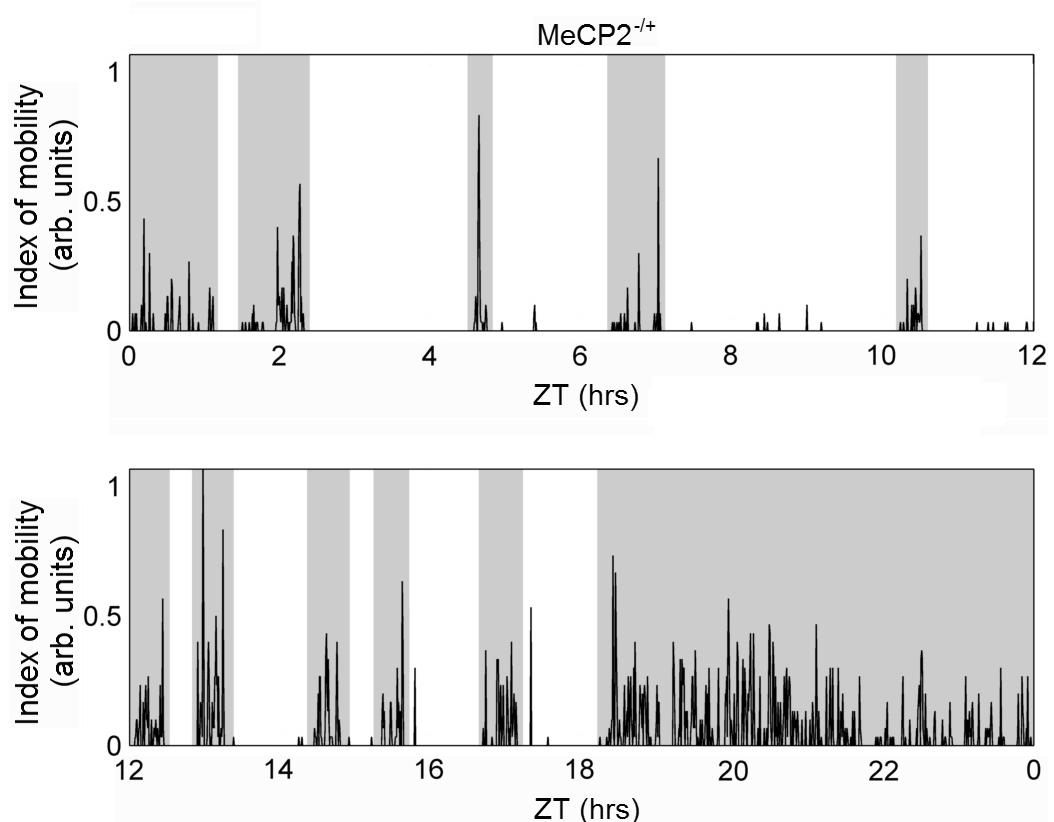
**D i)**

**ii)**

**Figure S2 Cont’d**


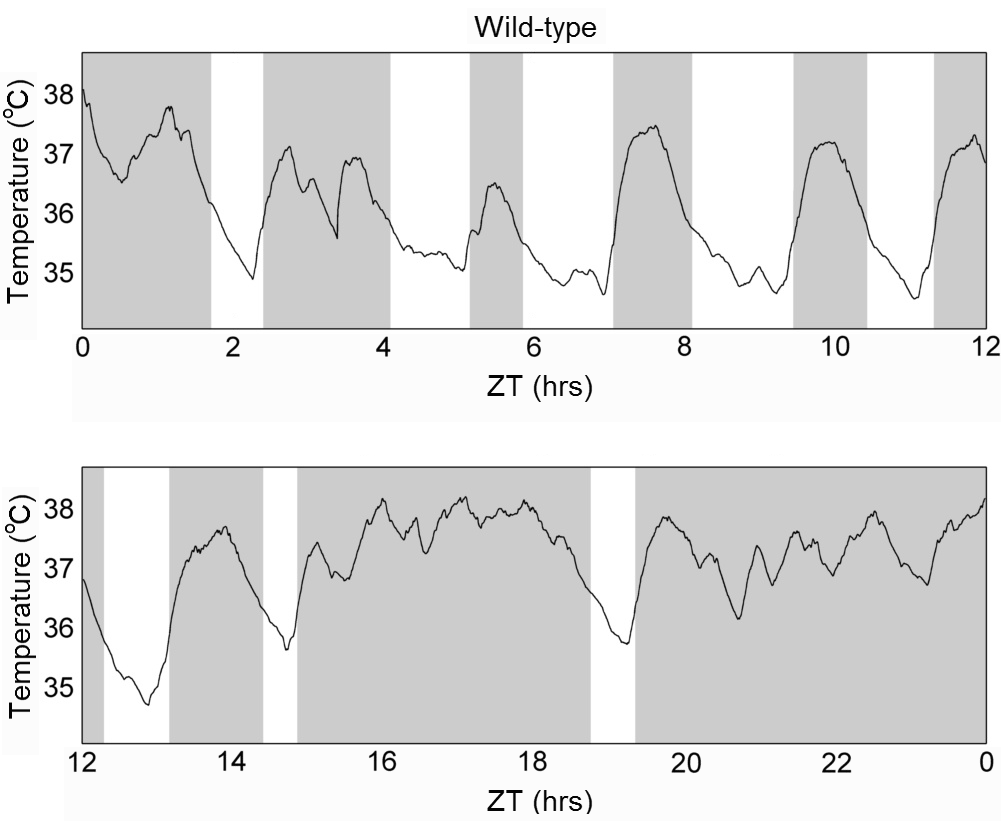
**E i)**

**ii)**


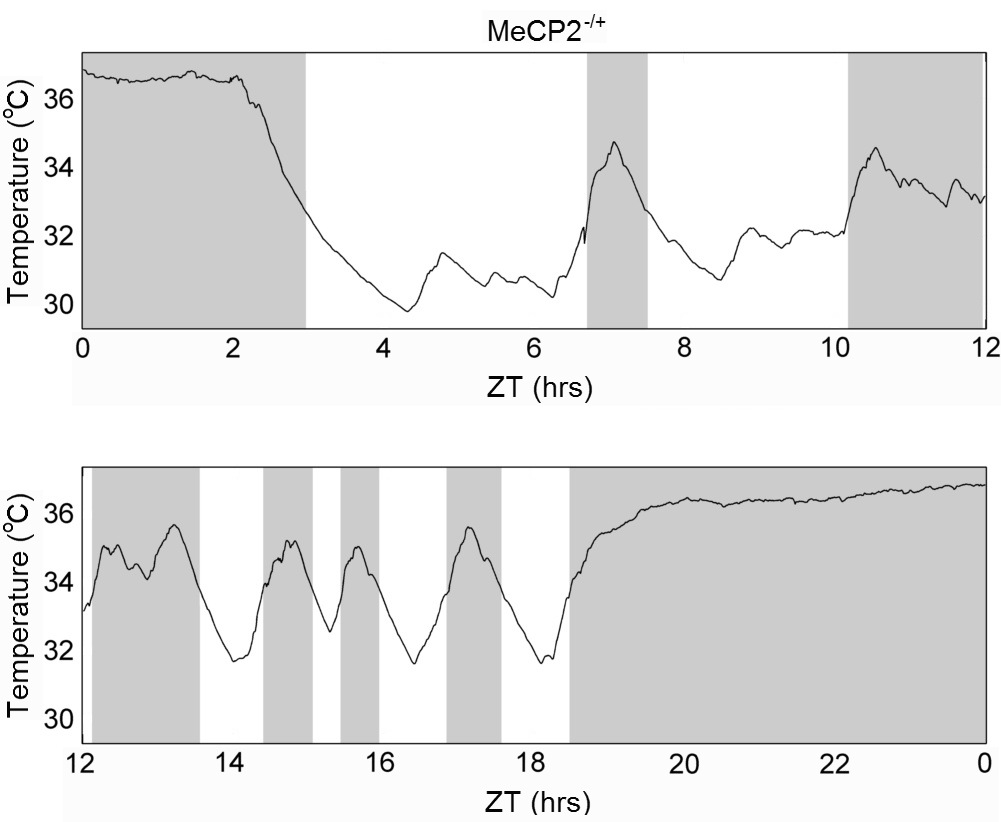
**F i)**

**ii)**

Supplement: Figure S2 — Recognition of periodic variations in EEG, gross motor activity, and core body temperature. Panels A and B: Representative traces of cortical delta power patterning over the light (i) and dark (ii) phases of a 24 hour day in a wild-type (A) and a MeCP2−/+ (B) mouse. Shaded regions denote areas classified as high delta states and non-shaded regions denote areas classified as low (non) delta states. Panels C and D: Representative traces of mobility patterning over a 24 hour day in a wild-type (C) and a MeCP2−/+ (D) mouse. Shaded regions denote areas classified as mobile behavioral states whereas non-shaded regions denote areas classified as inactive behavioral states. Panels E and F: Representative traces of core body temperature patterning over a 24 hour day in a wild-type (E) and a MeCP2−/+ (F) mouse. Shaded regions denote areas where body temperature was above the daily mean value, whereas non-shaded regions denote areas where body temperature was below the mean. (DOC) [file pone.0035396.s003.doc]

**Figure S3**


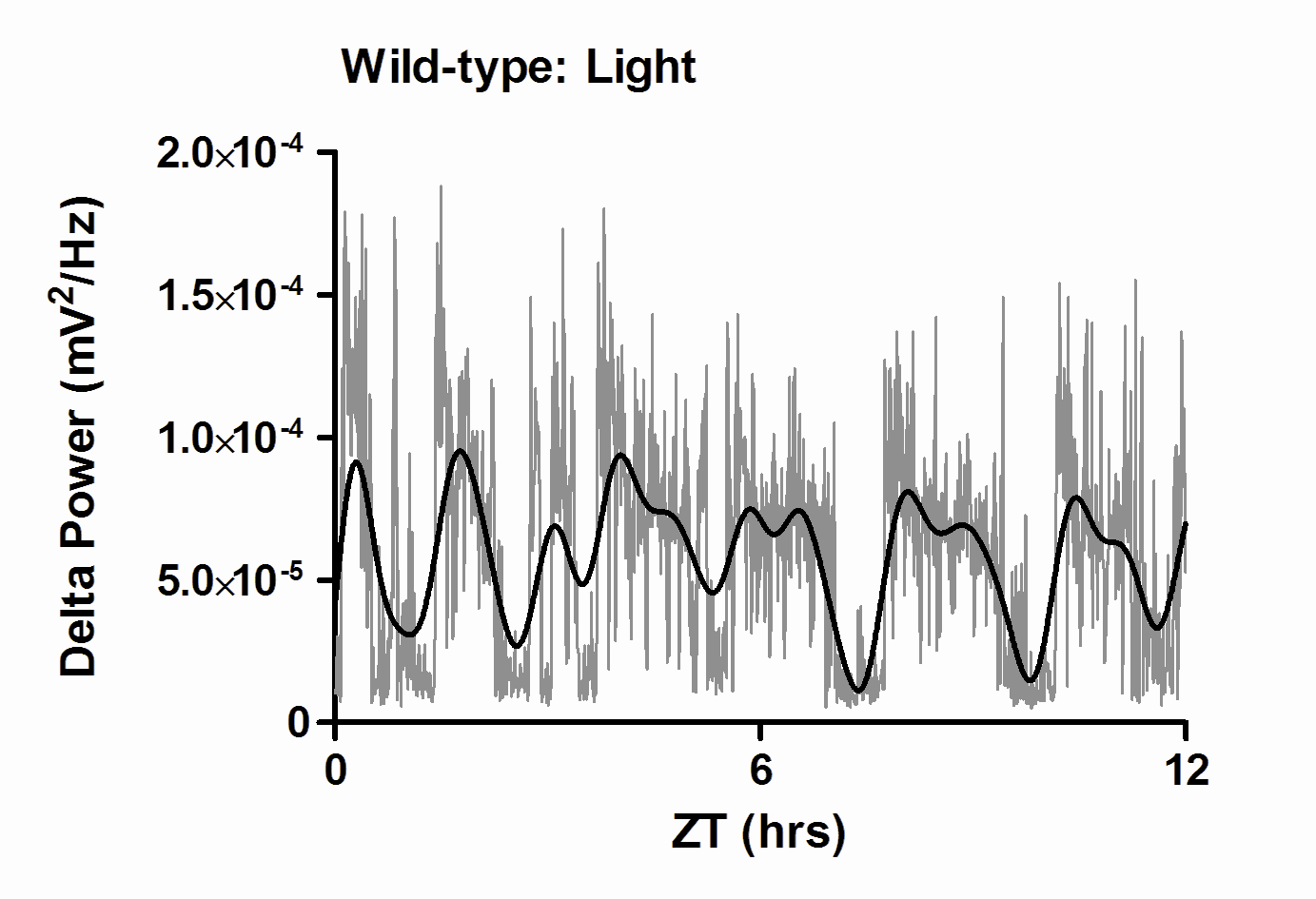

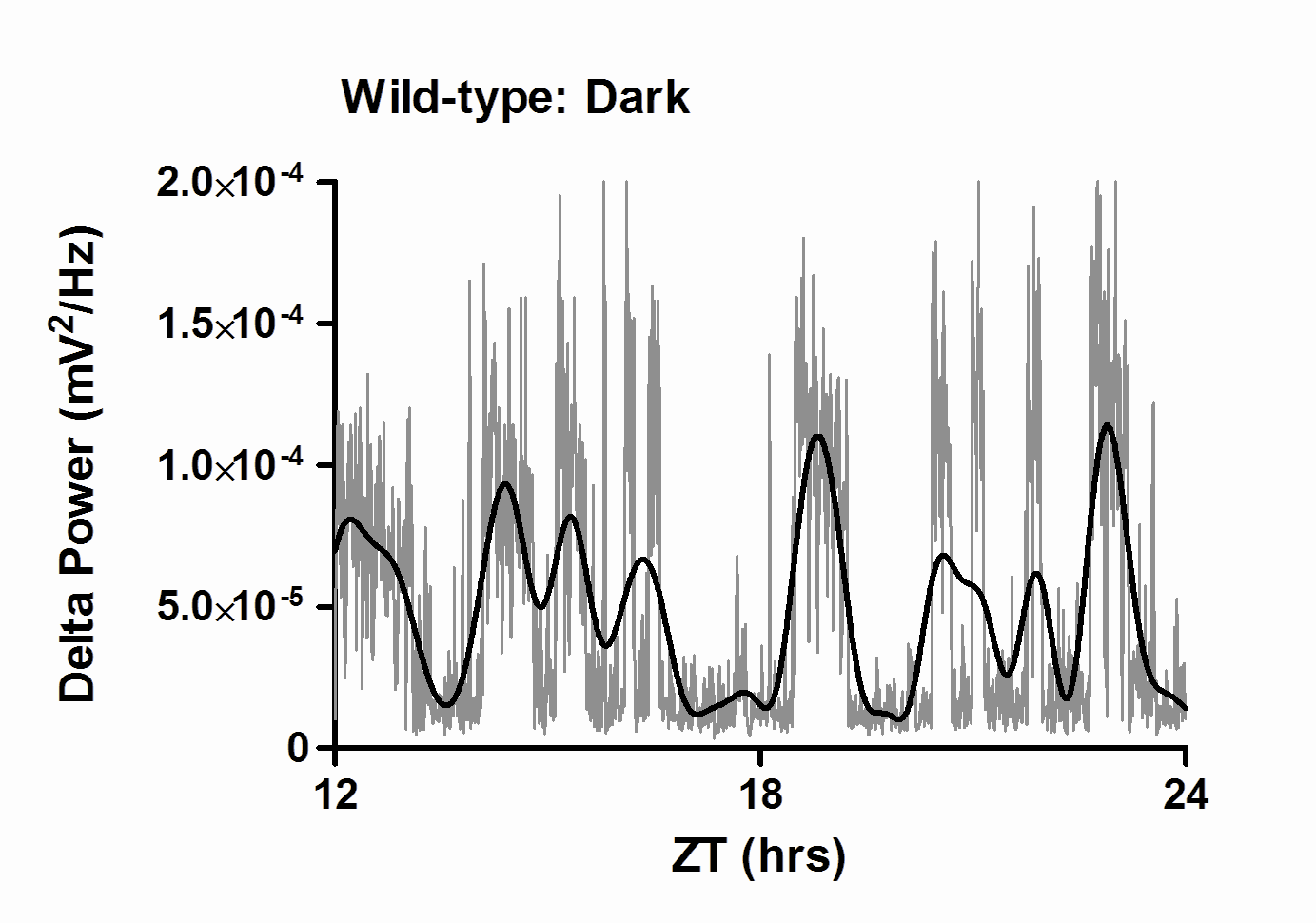
**A B**


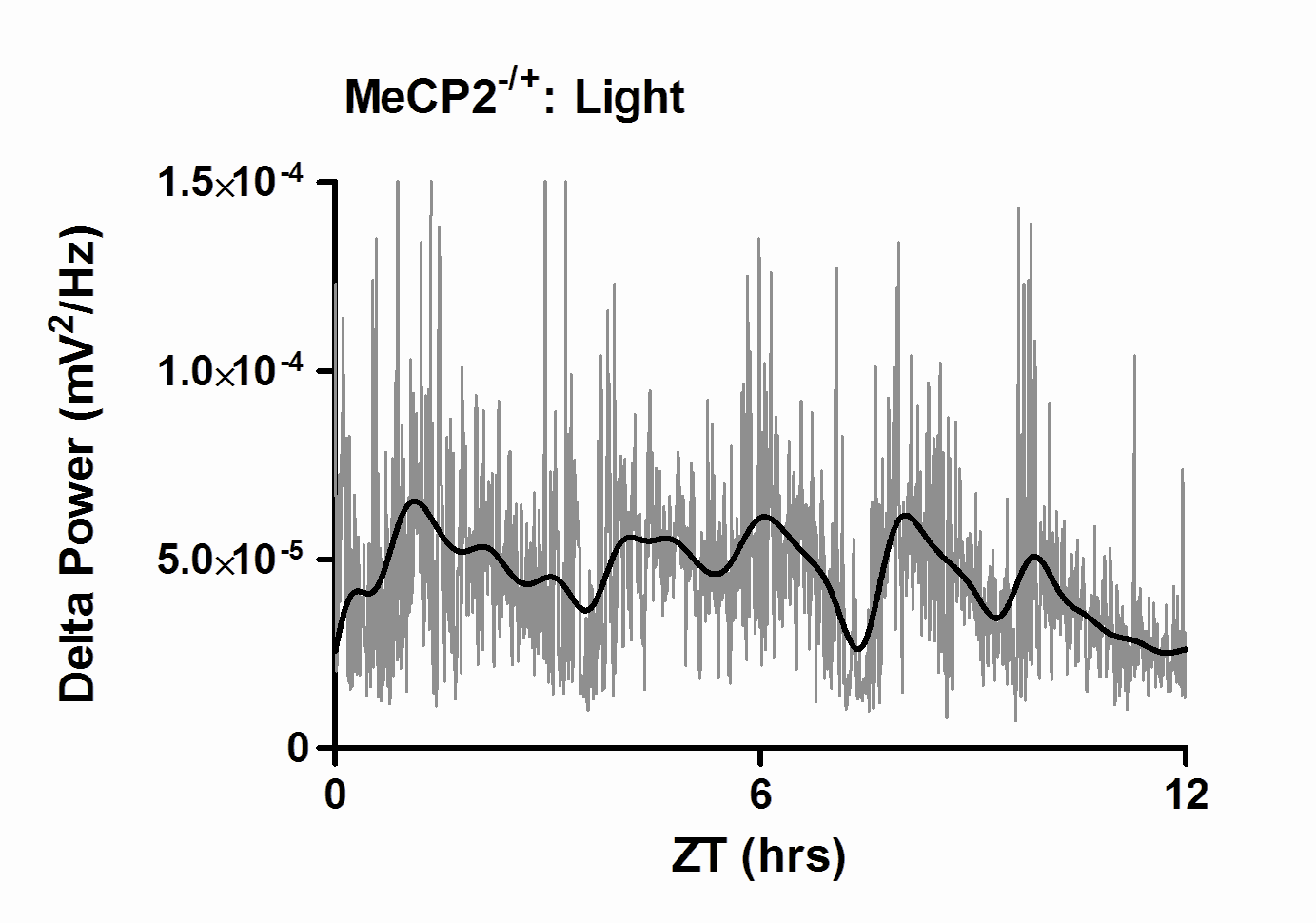

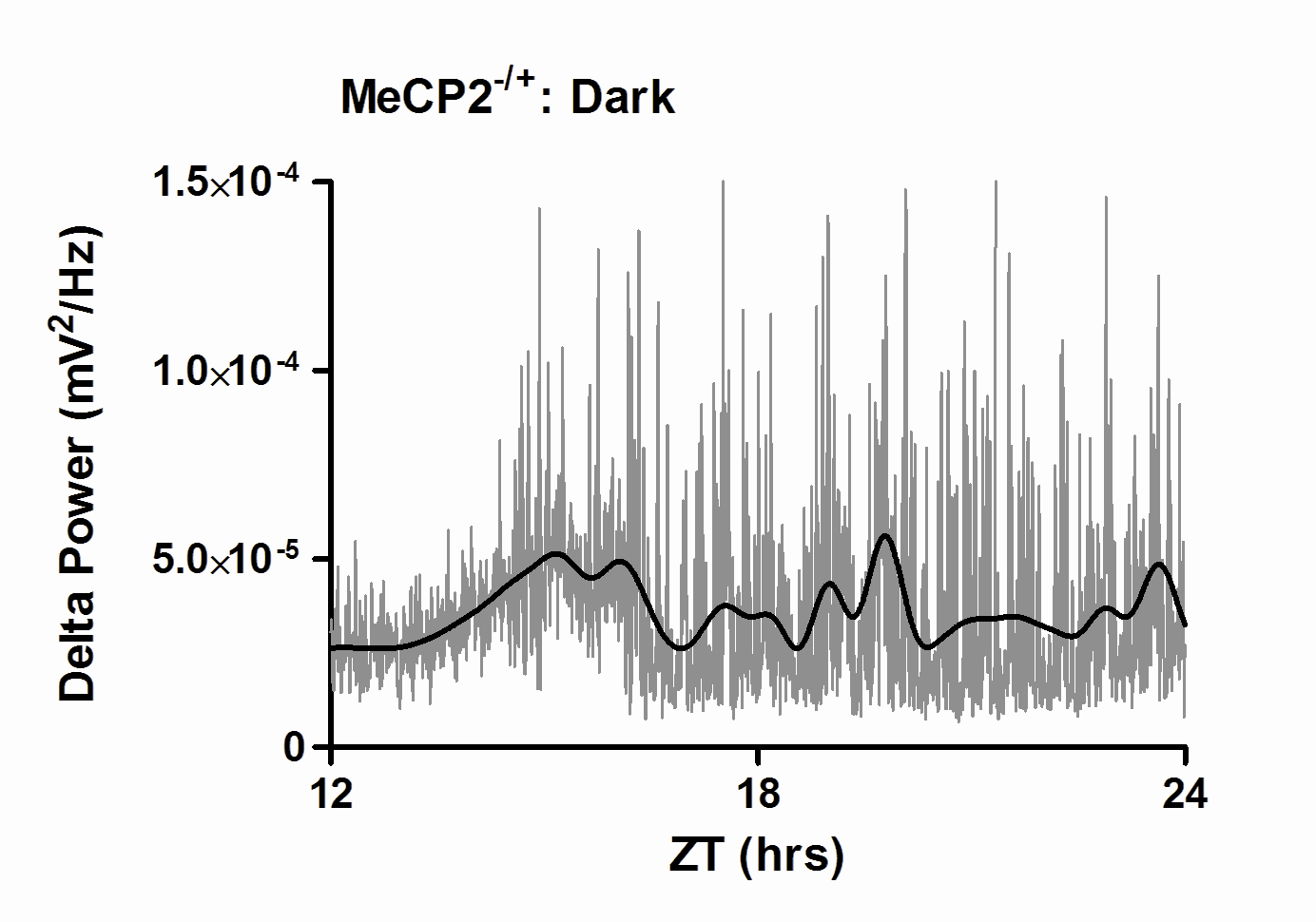
**C D**


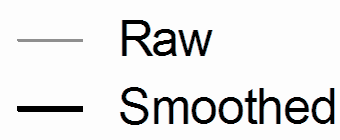


**Figure S3 Cont’d**


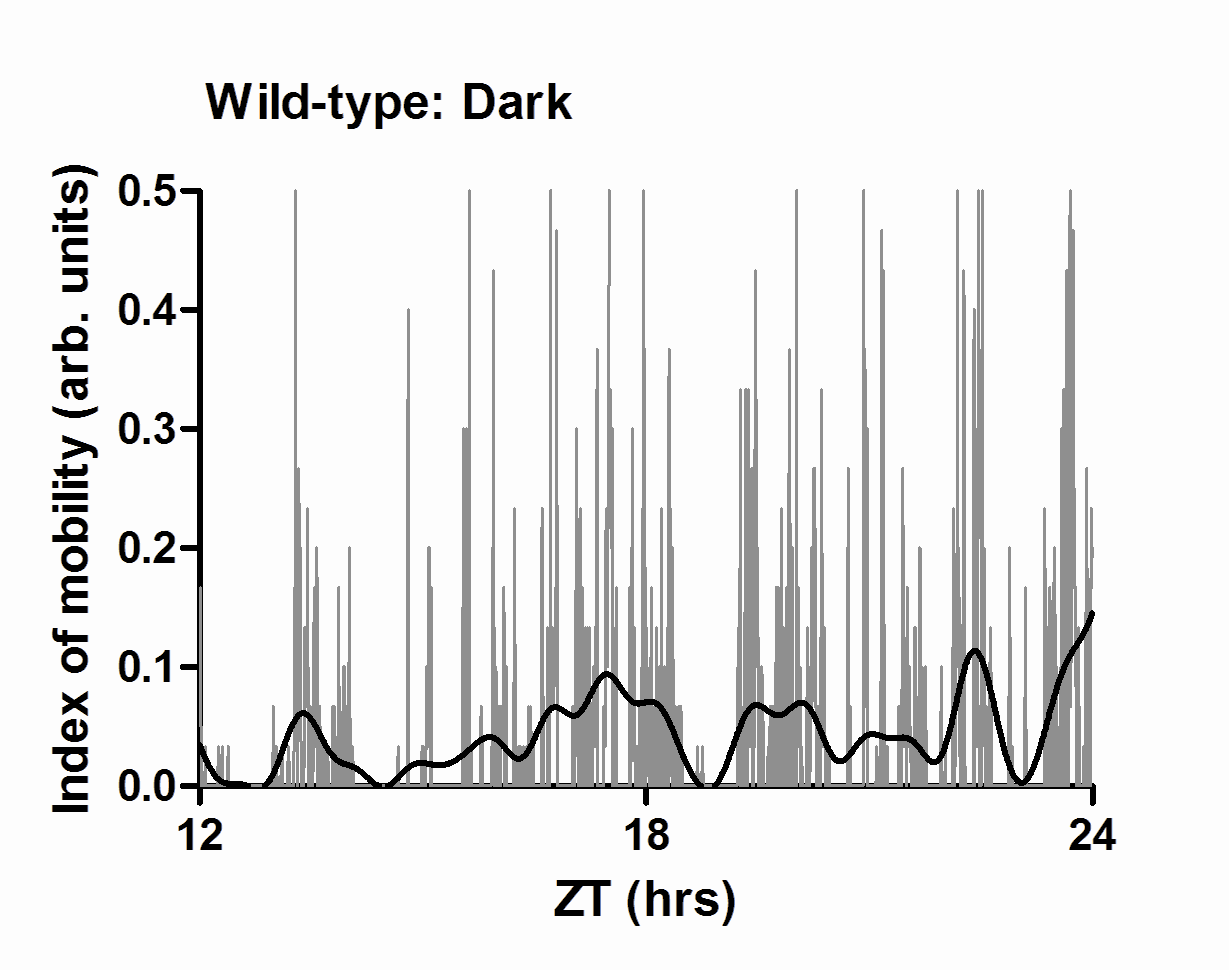

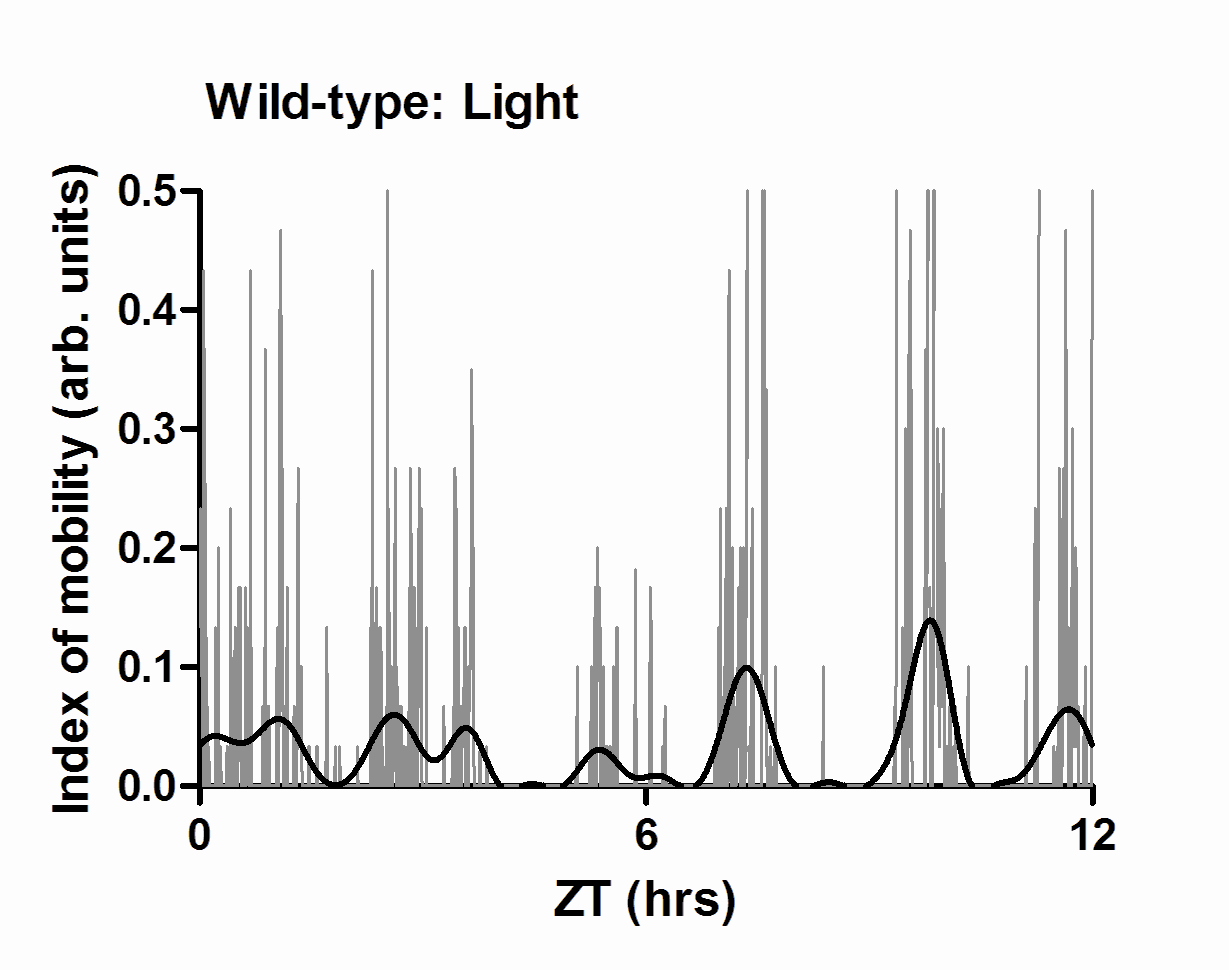
**E F**


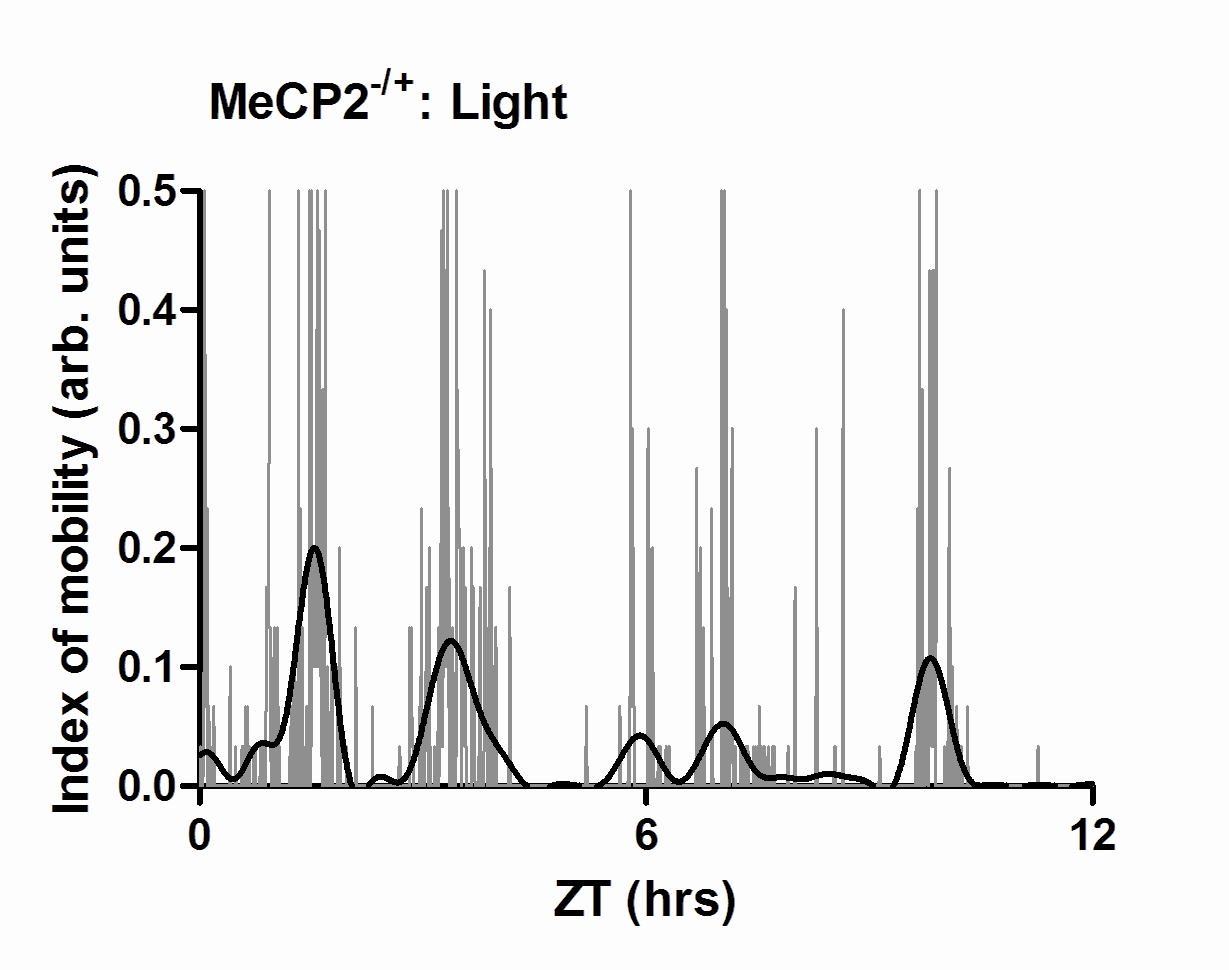

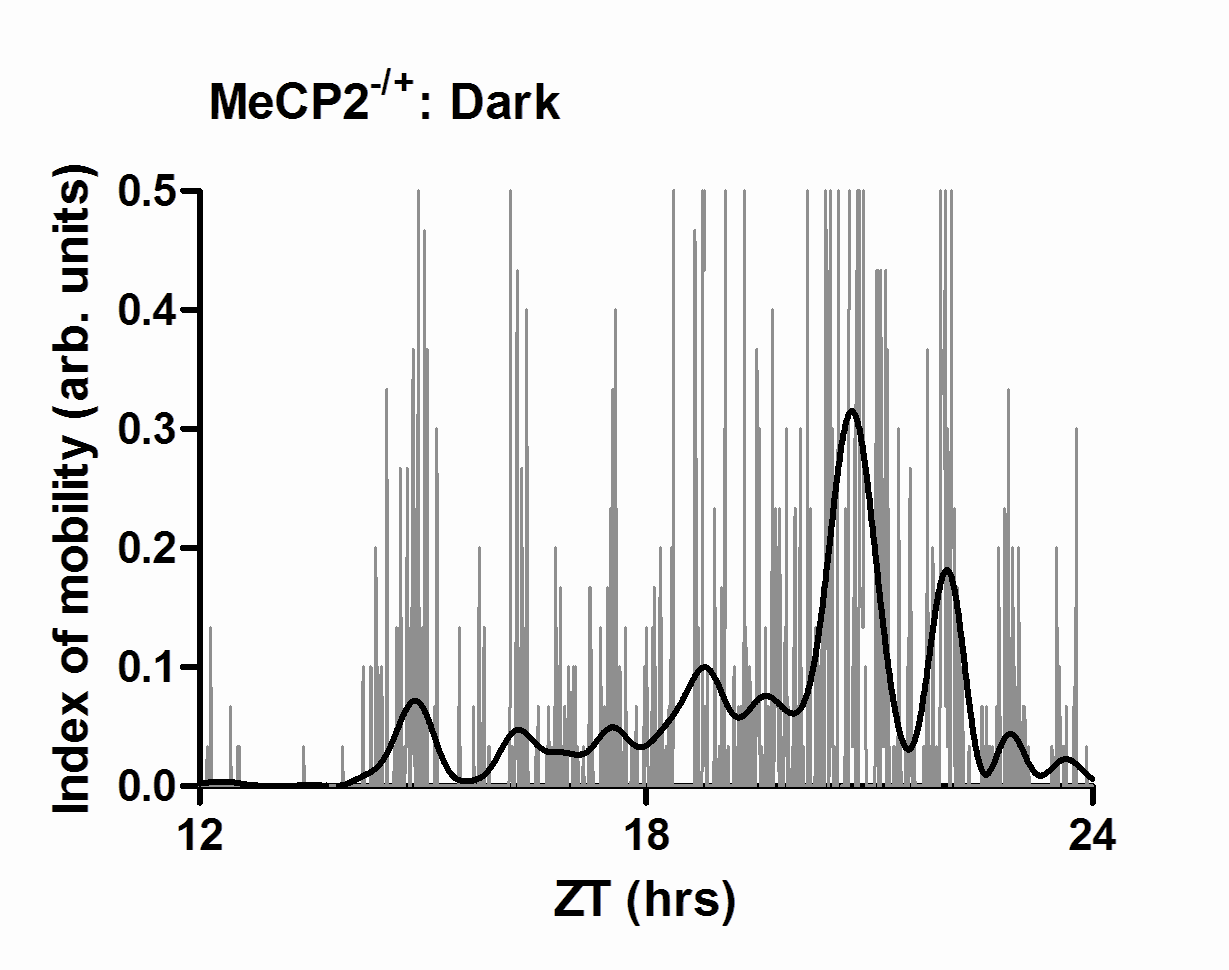
**G H**

**Figure S3 Cont’d**


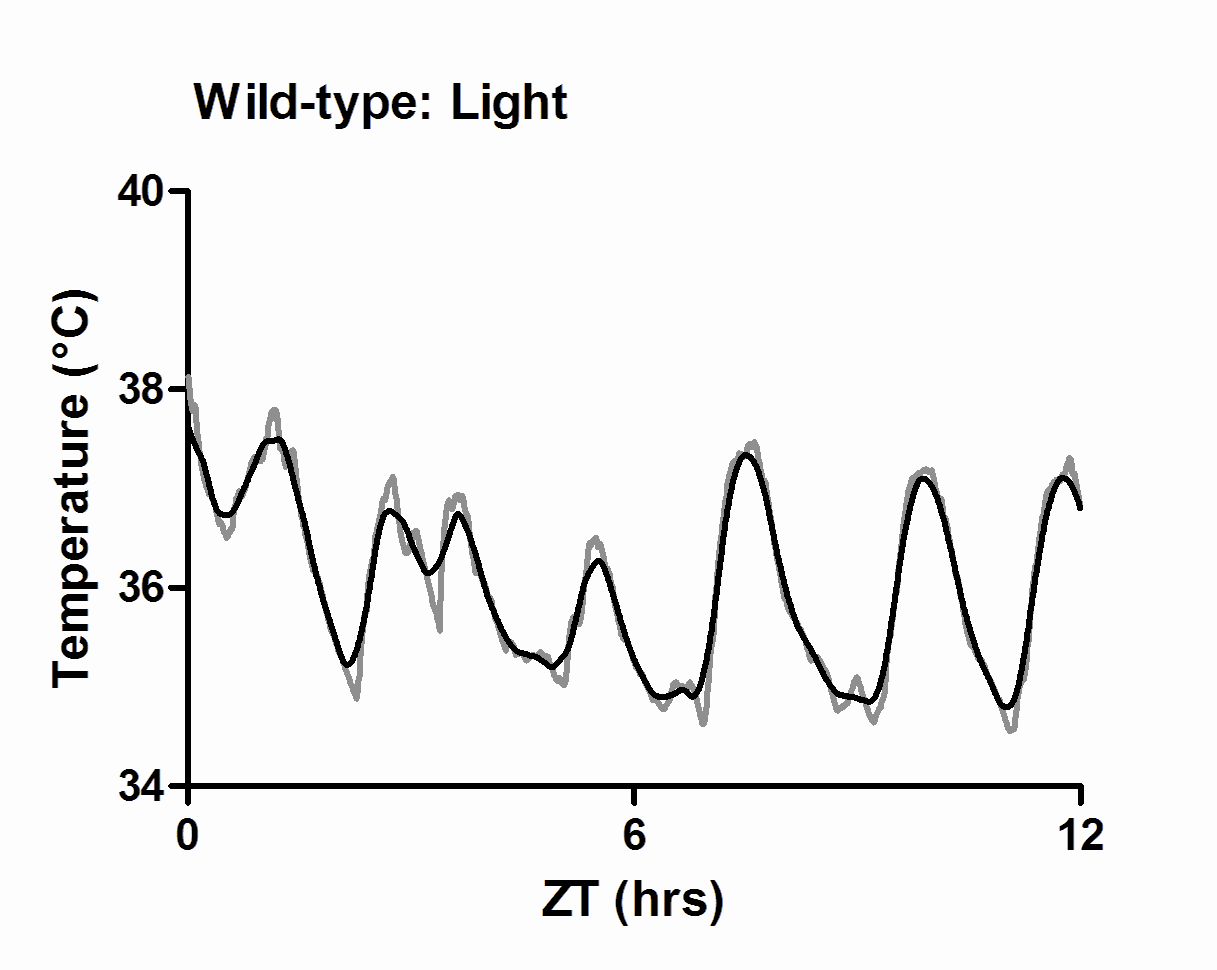

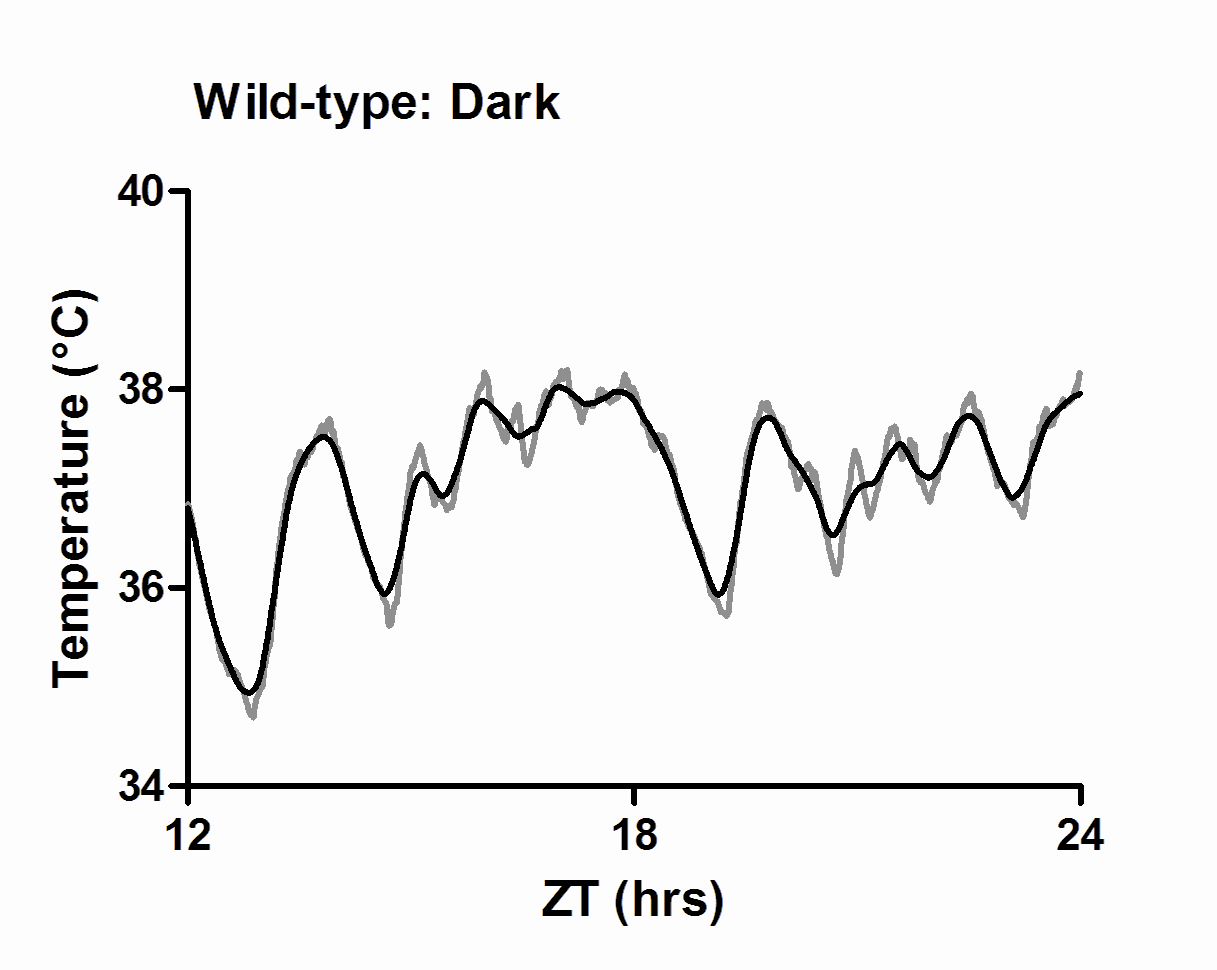
**I** **J**


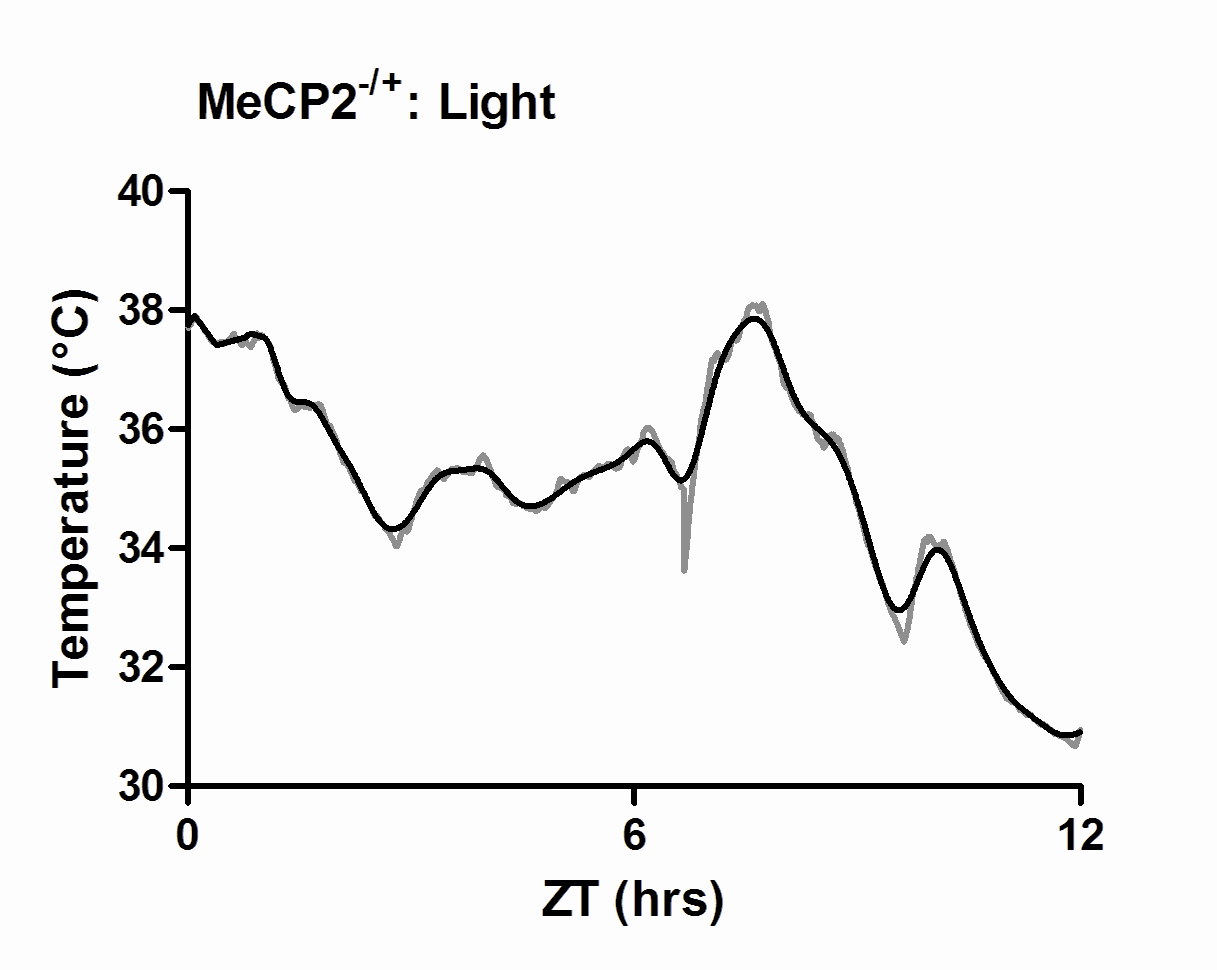

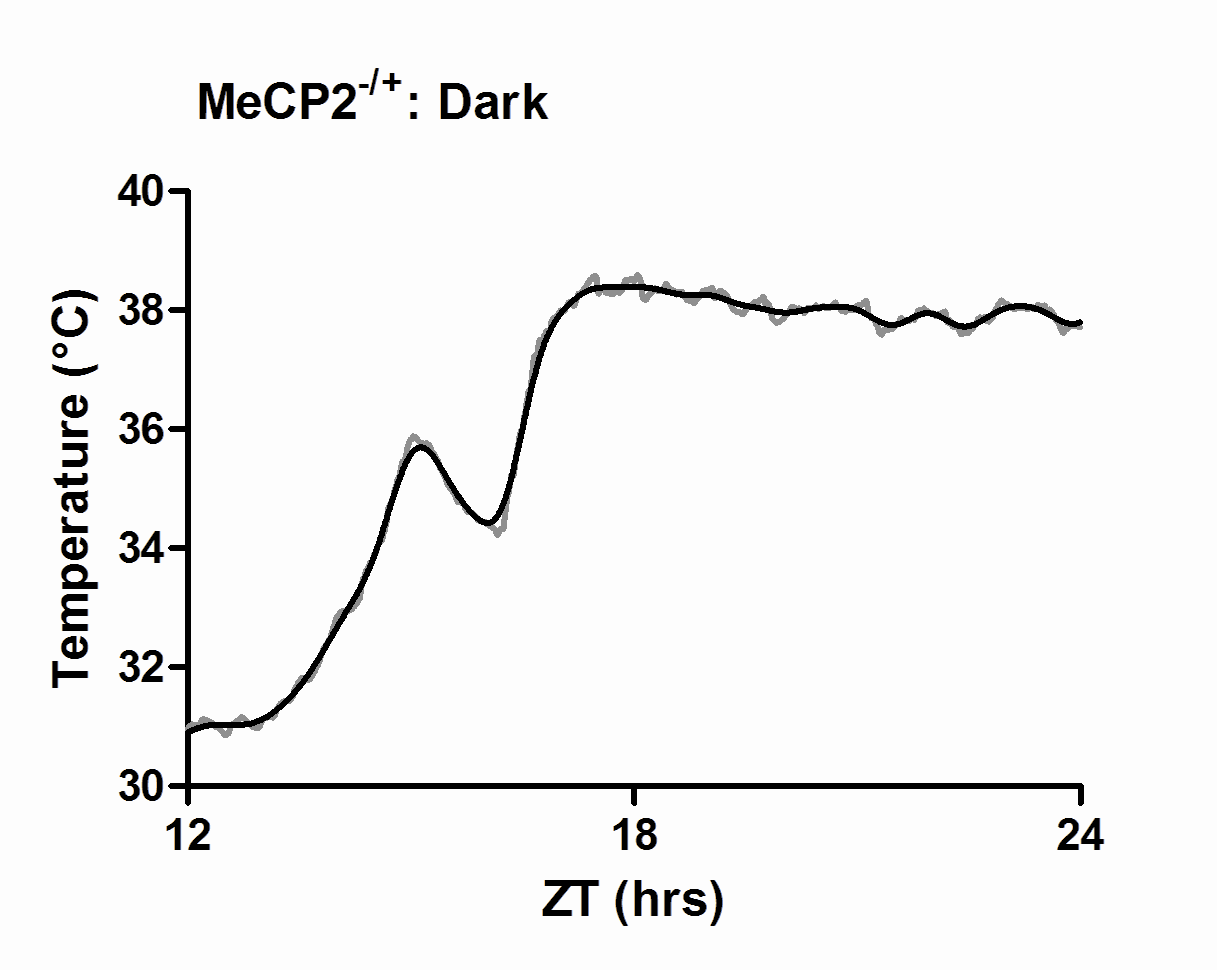
**K L**

Supplement: Figure S3 — Illustration of smoothed data generated from raw delta power, mobility, and body temperature traces. Panels A–D: Representative traces of raw cortical delta power (grey line) and the resulting smoothed data (black line) as generated using the 50-point Fast Fourier Transformation (FFT) smoothing function in OriginPro 6.1 (OriginLab Corporation, Northampton, MA) for a wild-type (A and B) and a MeCP2−/+ (C and D) mouse during the day (A and C) and night (B and D) phases of the 24-hour day. Panels E–H: Representative traces of raw mobility (grey line) and the resulting smoothed data (black line) as generated above for a wild-type (E and F) and a MeCP2−/+ (G and H) mouse during the day (E and G) and night (F and H) phases of a 24-hour day. Panels I–L: Representative traces of raw core body temperature (grey line) and the resulting smoothed data (black line) as generated above for a wild-type (I and J) and a MeCP2−/+ (K and L) mouse during the day (I and K) and night (J and L) phases of a 24-hour day. (DOC) [file pone.0035396.s004.doc]
